# A single-plasmid approach for genome editing coupled with long-term lineage analysis in chick embryos

**DOI:** 10.1242/dev.193565

**Published:** 2021-04-15

**Authors:** Shashank Gandhi, Yuwei Li, Weiyi Tang, Jens B. Christensen, Hugo A. Urrutia, Felipe M. Vieceli, Michael L. Piacentino, Marianne E. Bronner

**Affiliations:** 1 Division of Biology and Biological Engineering, California Institute of Technology, Pasadena, CA 91125, USA; 2 Department of Neuroscience, University of Copenhagen, Copenhagen, Blegdamsvej 3B, 2200 Copenhagen N, Denmark

**Keywords:** CRISPR-Cas9, Chick embryology, Clonal analysis, Retroviruses, Migration, Genome editing, Neural crest

## Abstract

An important strategy for establishing mechanisms of gene function during development is through mutation of individual genes and analysis of subsequent effects on cell behavior. Here, we present a single-plasmid approach for genome editing in chick embryos to study experimentally perturbed cells in an otherwise normal embryonic environment. To achieve this, we have engineered a plasmid that encodes Cas9 protein, gene-specific guide RNA (gRNA), and a fluorescent marker within the same construct. Using transfection- and electroporation-based approaches, we show that this construct can be used to perturb gene function in early embryos as well as human cell lines. Importantly, insertion of this cistronic construct into replication-incompetent avian retroviruses allowed us to couple gene knockouts with long-term lineage analysis. We demonstrate the application of our newly engineered constructs and viruses by perturbing β-catenin *in vitro* and *Sox10*, *Pax6* and *Pax7* in the neural crest, retina, and neural tube and segmental plate *in vivo*, respectively. Together, this approach enables genes of interest to be knocked out in identifiable cells in living embryos and can be broadly applied to numerous genes in different embryonic tissues.

## Abstract (precis)

**Summary:** A single-plasmid approach using self-cleaving ribozymes enables delivery of CRISPR components via electroporation and can be integrated into viral delivery systems for long-term tracking of mutant cells.

## INTRODUCTION

Advent of CRISPR-Cas9 (clustered regularly interspaced short palindromic repeats-CRISPR-associated protein 9) is a highlight of the past decade that has enabled genome editing across multiple species (Chen et al., 2013; Cong et al., 2013; Dickinson et al., 2013; Gagnon et al., 2014; Stolfi et al., 2014). Recently, it has been applied to early gene perturbations at stages ranging from gastrulation to neurulation in the chick embryo (Gandhi et al., 2017; Williams et al., 2018), an important model organism owing to its amenability to transplantation, surgical ablation, live imaging and gene perturbation (Darnell and Schoenwolf, 2000; Li et al., 2019; Streit et al., 2013).

Conventional molecular perturbation studies in chick embryos have relied upon electroporation of dominant-negative transgenes, antisense morpholinos or short-hairpin RNAs (Corey and Abrams, 2001; Sauka-Spengler and Barembaum, 2008) and, more recently, Cas9 and guide RNAs (gRNAs) (Gandhi et al., 2017; Williams et al., 2018). A major drawback of the latter approach was that Cas9 and gRNAs were delivered through separate constructs. Although only those cells co-transfected with both plasmids were mutants, these could not be distinguished from cells transfected with the fluorescent marker alone. In addition, the transient nature of electroporation resulted in the dilution of transcripts and encoded proteins over time (Andreason and Evans, 1988; Eisen and Smith, 2008; Nakamura and Funahashi, 2001). As a result, knocking out genes expressed at later embryonic stages was challenging, particularly in tissues that are not easily amenable to electroporation. Moreover, the absence of permanent labeling compromised the ability to identify or trace the descendants of individually perturbed cells.

Here, we report several improvements to the CRISPR-Cas9 toolkit for the generation of fluorescently labeled mutant cells in different tissues of the chick embryo. First, we use a self-cleaving ribozyme-mediated approach to facilitate production of the optimized gRNA^f+e^ scaffold (Chen et al., 2013; Gandhi et al., 2017) through an RNA polymerase (RNA-pol) II-based promoter. By eliminating the need for a chick U6 promoter to drive gRNA transcription, this strategy allows delivery of Cas9 and gRNA via a single plasmid. Second, we incorporate this ribozyme gRNA downstream of Cas9 flanked with nuclear localization signal sequences followed by a fluorescent protein (Williams et al., 2018) to enable fluorescent readout of mutant cells. This single-plasmid approach was used to knock out β-catenin *in vitro* in multiple species. Next, we demonstrate the ability to knock out *Sox10* and *Pax7* in the neural crest, and *Pax6* in the optic vesicle and hindbrain of chicken embryos using electroporation, and the ability to analyze the movement of *Sox10* mutant cells through live imaging. Finally, we enable cistronic integration of Cas9, Citrine and gene-specific gRNA in the host genome by constructing replication-incompetent avian RIA-CRISPR retroviruses. We demonstrate that virally infected identifiable cells can be successfully perturbed, labeled, and their progeny tracked long-term. Taken together, our approach combines the targeted-knockout ability of CRISPR-Cas9 with retroviral delivery to establish a method that enables genetic perturbations of identifiable mutant cells in an otherwise normal microenvironment of both early- and late-stage chick embryos, and potentially other species.

## RESULTS AND DISCUSSION

### A single-plasmid approach to deliver CRISPR components

Our previously reported modifications in designing individual CRISPR-Cas9 constructs substantially improved gene editing in early chick embryos (Gandhi et al., 2017), allowing successful gene knockouts in numerous studies following electroporation in early embryos (Gandhi et al., 2020a; Hutchins and Bronner, 2018; Piacentino and Bronner, 2018; Tani-Matsuhana et al., 2018; Vieceli and Bronner, 2018). However, because Cas9 and gRNAs were delivered via separate constructs, a complication was that only cells co-transfected with Cas9 and gRNA plasmids were mutants.

**Fig. 1.**
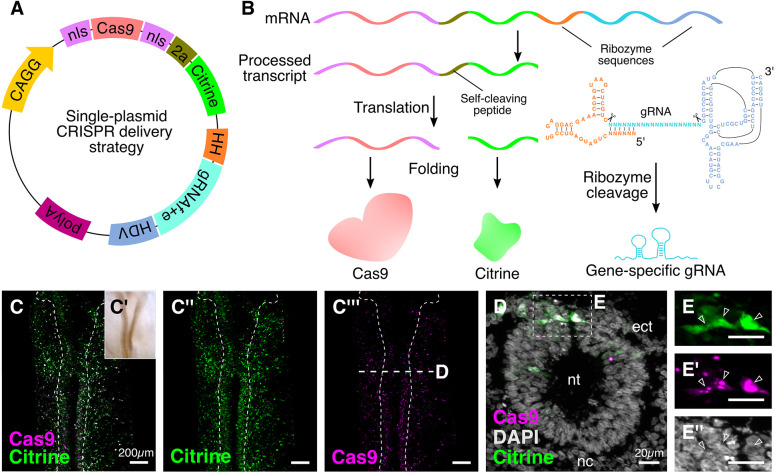
**Optimizing delivery of CRISPR components in chick embryos.** (A) A single-plasmid engineered vector for optimal delivery of Cas9, gene-specific gRNA, and the fluorescent protein Citrine in transfected cells. (B) After transfection, the cistronic transcript is cleaved at the HH and HDV ribozyme sites, thereby releasing the gene-specific gRNA. Following translation of the remaining transcript, Cas9 and Citrine are separated through self-cleavage of the 2A peptide. (C-C‴) Embryos electroporated with the plasmid depicted in A at HH9 (C′) and stained with antibodies against Citrine (C″) and Cas9 (C‴) to verify cellular localization. Dashed outline delineates the neural tube. (D) A transverse cross-section through the embryo shown in C, at the indicated level, with DAPI-stained nuclei. (E-E″) Enlarged views of the boxed area in D, showing that Citrine was enriched in the cytoplasm (E, arrowheads), whereas Cas9, in the absence of a gene-specific gRNA, was restricted to the nucleolus (E′, arrowheads) within the DAPI-stained nuclei (E″). ect, ectoderm; nc, notochord; nt, neural tube.

To circumvent this limitation and facilitate delivery of all components through a single construct, we explored alternative strategies to transcribe gRNAs that eliminated the requirement for a U6 promoter, thereby enabling Cas9 and gRNA production via the same promoter. We turned to a ribozyme-based approach (Gao and Zhao, 2014; He et al., 2017), flanking the gRNA^f+e^ backbone with the hammerhead (HH) ribozyme on the 5′ end, and the hepatitis delta virus (HDV) ribozyme on the 3′ end (Fig. 1A). Once transcribed, the precursor *HH:gRNA^f+e^:HDV* transcript undergoes self-catalyzed cleavage to release a functional gRNA molecule, allowing Cas9-mediated genome targeting (Fig. 1B). As a marker for successful transfection, we also included Citrine, which was separated from Cas9 by the self-cleaving 2A peptide sequence as described by Williams et al. (2018).

We tested this cistronic system by cloning the *HH:ControlgRNA^f+e^:HDV* fragment downstream of *Cas9-2A-Citrine* under regulation of the ubiquitous chicken β-actin promoter (Fig. 1A). We electroporated gastrula-stage chick embryos with this construct, cultured them *ex ovo* until Hamburger and Hamilton stage (HH) 9 (Fig. 1C′), and then processed them for immunostaining against Cas9 and Citrine (Fig. 1C-C‴). As expected, Citrine (Fig. 1C″) and Cas9 (Fig. 1C‴) were expressed in transfected cells throughout the embryo. Transverse sections further validated the respective localizations of individual components (Fig. 1D-E″), with Citrine expression in the cytoplasm (Fig. 1E), and Cas9 expression in the nucleus (Fig. 1E′) overlapping with that of DAPI (Fig. 1E″). In fact, Cas9 was retained in the nucleolus, consistent with previous work that demonstrated this phenomenon when Cas9 is not associated with a gene-specific gRNA (Gu et al., 2018). Together, these results indicate that addition of the *HH:gRNA^f+e^:HDV* precursor does not interfere with the nuclear and cytoplasmic localization of Cas9 and the fluorescent protein, respectively.

### The single-plasmid strategy can efficiently perturb function across species

**Fig. 2.**
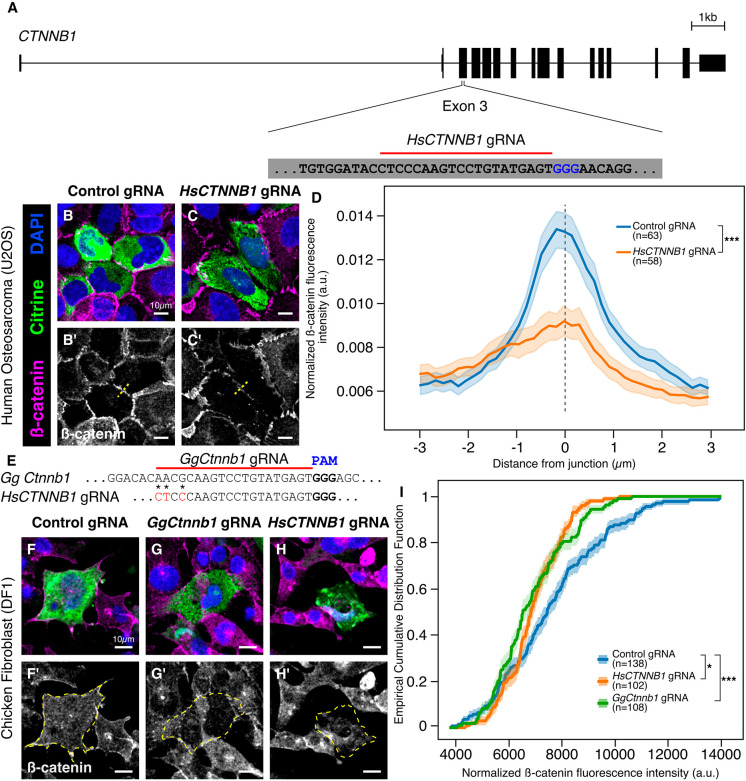
**A** **single CRISPR plasmid efficiently perturbs function across multiple species.** (A) The genomic locus for *CTNNB1* in the human genome. A gRNA protospacer targeting the third exon was designed. (B-C′) Representative images from human epithelial cells following transfection with the single CRISPR plasmid harboring control gRNA (B,B′) and human *CTNNB1*-targeted gRNA (C,C′). (D) Mean fluorescence intensity from line scan analysis (dashed lines in B′,C′) shows that junctions between transfected cells display reduced β-catenin immunoreactivity following *CTNNB1* knockout [Kolmogorov–Smirnov test, ***P<0.001, *n*=63 (control gRNA) and *n*=58 (*CTNNB1* gRNA)]. (E) Sequence alignment surrounding the *Ctnnb1* gRNA protospacer between human and chicken. Red characters within the *HsCTNNB1* gRNA protospacer indicate non-conserved nucleotides (asterisks). (F-H′) Representative images from chicken fibroblast cell culture following transfection with the single CRISPR plasmid harboring control gRNA (F,F′), chicken *Ctnnb1*-targeted gRNA (G,G′) and human *CTNNB1*-targeted gRNA (H,H′). Dashed yellow line in F′-H′ represents the cell boundary that was used to calculate cell fluorescence. (I) Empirical cumulative distribution frequency analysis indicates that gRNAs targeting human or chicken *Ctnnb1* cause significant reduction of β-catenin immunolabeling within chicken fibroblasts compared with non-binding control gRNA transfections [Kruskal–Wallis test, *P<0.05, **P<0.01, *n*=138 (control gRNA), *n*=102 (*HsCTNNB1* gRNA), *n*=108 (*GgCtnnb1* gRNA)]. a.u., arbitrary units. Shaded areas in D and I represent s.e.m.

To validate that the *HH:gRNA^f+e^:HDV* precursor cleaves properly to release a functional gRNA molecule, we turned to an *in vitro* transfection-based approach, targeting the β-catenin (*CTNNB1*) locus in human epithelial osteosarcoma U2OS cells (Fig. 2A). We identified a protospacer in the third exon of the *CTNNB1* coding sequence and produced the plasmid *CAG>nls-Cas9-nls-2A-Citrine-HH-HsCTNNB1gRNA^f+e^-HDV* to knock out β-catenin. We transfected U2OS cells with this plasmid, incubated them for 24 h at 37°C, and then fixed and processed them for immunostaining against β-catenin. Cells in the ‘control’ well were transfected with the *CAG>nls-Cas9-nls-2A-Citrine-HH-CtrlgRNA^f+e^-HDV* plasmid. Whereas β-catenin was retained at the cellular junctions of control cells (Fig. 2B,B′), those successfully electroporated with the *CTNNB1gRNA*-containing construct, as identified by the expression of Citrine, had reduced levels of β-catenin at cell junctions (Fig. 2C,C′). β-Catenin fluorescence intensity across cell junctions was quantified by line scan analysis, with transfected cells displaying a significant reduction in β-catenin protein production following *CTNNB1* knockout compared with those transfected with non-binding control gRNA construct (Fig. 2D; *P*<0.001, Kolmogorov–Smirnov test). This confirms that *CTNNB1* gRNA molecules were successfully synthesized in transfected human U2OS cells, and this single plasmid was sufficient to reduce β-catenin protein levels.

We next investigated whether our single-plasmid approach would allow CRISPR-Cas9-mediated gene knockout in multiple species using the same single construct. To do this, we looked for homology between the human and chicken *Ctnnb1* genomic locus and discovered an 85% conservation between these species in the third exon. Importantly, the gRNA protospacer that allowed a robust β-catenin knockdown in human U2OS cells was highly conserved between the two species, with only three nucleotide differences distal to the PAM sequence (Fig. 2E, asterisks). Therefore, we tested the ability of our single-plasmid strategy to knock out *Ctnnb1* in chicken fibroblast cells (DF-1) using both human and chicken gRNA sequences. To this end, we transfected chicken DF-1 fibroblasts with the *CAG>nls-Cas9-nls-2A-Citrine-HH-HsCTNNB1gRNA^f+e^-HDV* plasmid, while transfecting *CAG>nls-Cas9-nls-2A-Citrine-HH-CtrlgRNA^f+e^-HDV* or *CAG>nls-Cas9-nls-2A-Citrine-HH-GgCtnnb1gRNA^f+e^-HDV* plasmids as negative and positive controls, respectively. We immunostained these cells against β-catenin 24 h post-transfection. Interestingly, both chicken (Fig. 2G,G′) and human (Fig. 2H,H′) plasmids were sufficient to reduce the levels of β-catenin compared with the control group (Fig. 2F,F′). We quantified this phenotype using cumulative distribution frequency analysis, which revealed significantly diminished β-catenin protein levels in transfected DF-1 cells when supplied with either the chicken or human gRNA constructs, compared with the non-binding control gRNA construct (Fig. 2I). That the same construct produced CRISPR-Cas9-mediated knockouts in both chick and human cells illustrates the versatility of our single-plasmid approach in performing perturbation experiments across species, especially when regions with high homology near the PAM site are targeted.

### Early effects of loss of *Sox10* using the single-plasmid approach

**Fig. 3.**
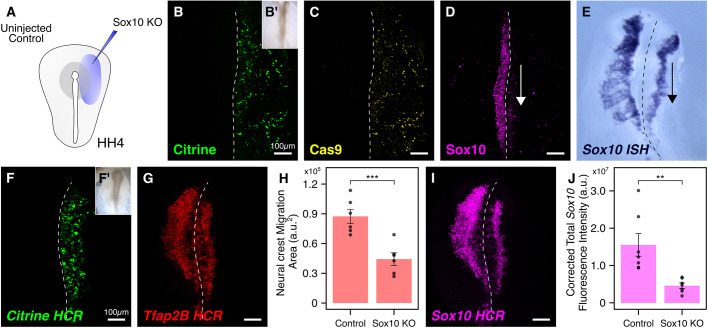
**Electroporation-mediated early knockout of *Sox10* using** **a** **single CRISPR plasmid.** (A) Electroporation strategy to target *Sox10* in the neural crest. The right side of gastrula-stage HH4 embryos was electroporated with a single CRISPR plasmid containing validated gRNA protospacer. (B-C) Embryos electroporated with the knockout construct developed to HH9 (B′) and immunostained for the expression of Citrine (B) and Cas9 (C). (D) The single-plasmid approach efficiently reduced Sox10 protein levels in emigrating cranial neural crest cells at HH9. (E) Chromogenic *in situ* hybridization (ISH) revealed a notable reduction in *Sox10* mRNA levels in migrating cranial neural crest cells on the knockout side at HH9+. (F-J) Knockout embryos (F′) were processed for *in situ* HCR against *Citrine* (F), *Tfap2B* (G) and *Sox10* (I). Quantification of migration area (H) and *Sox10* fluorescence intensity (J) showed significant reduction in total area occupied by cranial neural crest cells (H; paired Student's *t*-test, ***P<0.001, *n*=6), and *Sox10* mRNA levels (J; paired Student's *t*-test, **P<0.01, *n*=7) following single-CRISPR-plasmid-mediated *Sox10* knockout. a.u., arbitrary units. Error bars represent s.e.m. Dashed line in image panels marks the midline.

Using the loss of a gene of interest as a readout for efficient gene knockout, we next used our single-plasmid strategy to target the pan-neural crest marker *Sox10*. An important member of the neural crest gene regulatory network, *Sox10* is crucial for proper specification, migration and differentiation of neural crest cells into mature neurons and glia of the peripheral nervous system (Martik and Bronner, 2017). To test if the modified single-plasmid strategy was sufficient to perturb *Sox10* levels in the neural crest, we electroporated the right side of gastrula-stage embryos with the *CAG>nls-Cas9-nls-2A-Citrine-HH-Sox10gRNA^f+e^-HDV* plasmid (Fig. 3A) encoding a *Sox10* protospacer previously shown to result in its efficient knockdown (Gandhi et al., 2017). After culturing embryos *ex ovo* until HH9 or HH9+ to capture premigratory (Fig. 3B′) and migratory (Fig. 3F′) neural crest cells, respectively, we processed them for immunohistochemistry and *in situ* hybridization. Antibody staining for Citrine, Cas9 and Sox10 revealed expression of both Citrine (Fig. 3B) and Cas9 (Fig. 3C) on the electroporated side, as well as a drastic reduction in Sox10 protein levels in emigrating neural crest cells (Fig. 3D). Moreover, *in situ* hybridization revealed a significant downregulation in *Sox10* mRNA levels in migrating neural crest cells (Fig. 3E). For quantification, we processed targeted embryos for high-resolution *in situ* hybridization chain reaction (HCR). We first labeled nascent Citrine transcripts (Fig. 3F) and observed a strong signal, confirming active transcription through the electroporated single CRISPR plasmid on the transfected side. Next, we labeled the transcripts for the neural crest marker *Tfap2B*, which allowed us to quantify the effect of reduced *Sox10* on neural crest migration. We observed a 50% reduction in the migration area (*n*=6), with cranial neural crest cells occupying an area of 87,336±7203 a.u.^2^ on the control side compared to 44,264±6224 a.u.^2^ on the treatment side (Fig. 3H; *P*<0.001, paired Student's *t*-test). Finally, consistent with chromogenic *in situ* hybridization, we observed a notable reduction in the levels of *Sox10* mRNA (Fig. 3I) on the knockout side. Quantification of the corrected total cell fluorescence (CTCF) intensity revealed a 70% reduction in *Sox10* intensity (*n*=7) between the control and knockout sides (Fig. 3J; *P*<0.01, paired Student's *t*-test). Together, these results demonstrate that the single-plasmid approach is efficient in perturbing the levels of *Sox10 in vivo*.

### Using the single-plasmid approach for live imaging

**Fig. 4.**
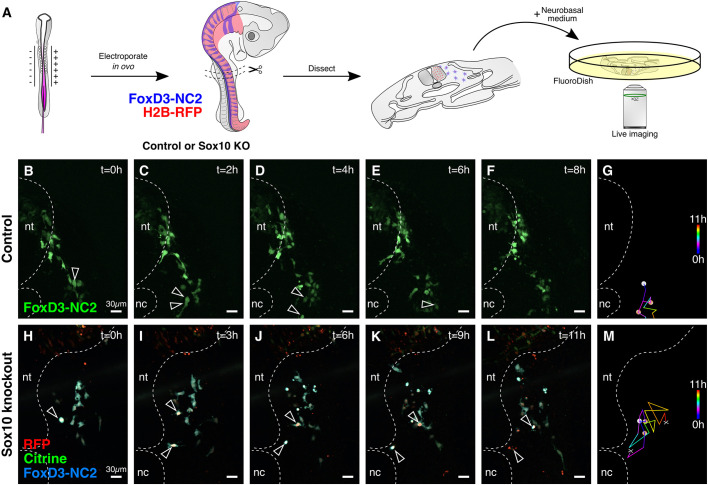
**Live imaging application of the single CRISPR plasmid.** (A) Strategy for processing control and mutant embryos for live imaging. Neural crest cells were labeled using the *FoxD3* enhancer NC2; knockout embryos were also electroporated with the single CRISPR plasmid targeting *Sox10* and H2B-RFP as a transfection marker. (B-F) In control embryos, trunk neural crest cells divide (B,C, arrowheads) and migrate properly (D-F), with the daughter cells leaving the imaging frame/plane in E and F. (G) Tracking migration of the cells highlighted in C reveals proper trunk neural crest migration. (H-L) When *Sox10* was knocked out before neural crest emigration, mutant cells still divided (H,I, arrowheads), but migrated in a circle (J,K) before undergoing apoptosis (L). (M) Cell tracking of mutant cells shows aberrant migration. Dashed line represents neural tube (nt) and notochord (nc).

Next, we explored the possibility of applying live imaging to analyze the effects of CRISPR-Cas9-mediated knockouts, which was not previously possible in chick cells because components were delivered using separate plasmids. To test this, we electroporated the neural tube of HH10 chick embryos with *FoxD3-NC2>Cerulean*, *CAG>H2B-RFP* and *CAG>nls-Cas9-nls-2a-Citrine-HH-Sox10gRNA-HDV* on the right side (Fig. 4A). Control embryos were electroporated with *FoxD3-NC2>GFP* at the same concentration. *FoxD3* is a neural crest specifier gene, expressed in premigratory and early migrating neural crest cells along the entire anterior-posterior axis (Stewart et al., 2006; Teng et al., 2008) of developing embryos. Its enhancer, NC2 (Simões-Costa et al., 2012), drives strong reporter expression in post-otic neural crest cells (Gandhi et al., 2020a; Ling and Sauka-Spengler, 2019), including the trunk neural crest (Simoes-Costa and Bronner, 2016). H2B-RFP was electroporated in knockout embryos as a nuclear transfection control to complement the cytoplasmic expression of Citrine. The embryos were cultured *in ovo* at 37°C for 24 h before harvesting. Using FoxD3-NC2-mediated reporter activity as a reference for neural crest expression, slice cultures were prepared spanning one or two somites in length (Li et al., 2020), which were then imaged for 11 h with a temporal resolution of 10 min.

To establish the baseline migration pattern of trunk neural crest cells as they migrated ventrally away from the dorsal neural tube toward the sensory and sympathetic ganglia, we visualized labeled control neural crest cells and their progeny (Fig. 4B). Control daughter cells after cell division migrated extensively and typically moved out of the imaging frame and/or plane during the imaging session (Fig. 4C-G, Movie 1). In contrast, labeled cells lacking *Sox10* exhibited aberrant cell migration (Fig. 4H-L, Movie 2). Although *Sox10* mutant cells still underwent mitosis (Fig. 4H,I), thus demonstrating that loss of *Sox10* did not inhibit their ability to divide, trajectory analysis revealed abnormal migration of the resultant sister cells before their apoptosis (Fig. 4M). These results are consistent with previous literature, in which loss of *Sox10* in premigratory neural crest resulted in premature apoptosis shortly after delamination (Dutton et al., 2001; Kelsh, 2006). Taken together, these results confirm that loss of *Sox10* prior to delamination of neural crest cells results in aberrant migration and premature apoptosis (Carney et al., 2006; Dutton et al., 2001), whereas its primary effects at later stages of neural crest development appear to be on neuronal and glial differentiation (Britsch et al., 2001). To our knowledge, this is the first study that combines CRISPR-Cas9-mediated knockouts with live imaging in chick embryos.

### Incorporating CRISPR components into RIA retroviruses

Although the electroporation strategy works well for gastrula- and neurula-stage embryos, many other tissues that form in late embryos are inaccessible to electroporation. Therefore, we investigated whether using viruses, which allow integration into the genome and ease of delivery, might represent an alternative method of introducing CRISPR-Cas9 components. In the chick embryo, RIA retrovirus has served as a versatile tool for long-term lineage tracing (Li et al., 2017, 2019; Tang et al., 2019). Once a susceptible cell is infected with this virus, the viral RNA is reverse transcribed and stably integrated into the host genome. Because the integrated viral DNA lacks the gene for the envelope protein to produce virus, it fails to horizontally transfer its genetic material into neighboring cells (Chen et al., 1999; Witte and Baltimore, 1977). However, as an intrinsic component of the host genome, the viral DNA can be vertically transferred to daughter cells through mitosis, resulting in permanent labeling of the entire lineage derived from infected progenitors. These combined attributes make RIA a powerful system for exploring lineage relationships within complex tissues (Li et al., 2017; Tang et al., 2019). Recently, this RIA-mediated approach has been extended for clonal analysis, by identifying rare color combinations coupled with intracellular localization of fluorophores that result from simultaneous infection of more than one virion (Tang et al., 2019).

**Fig. 5.**
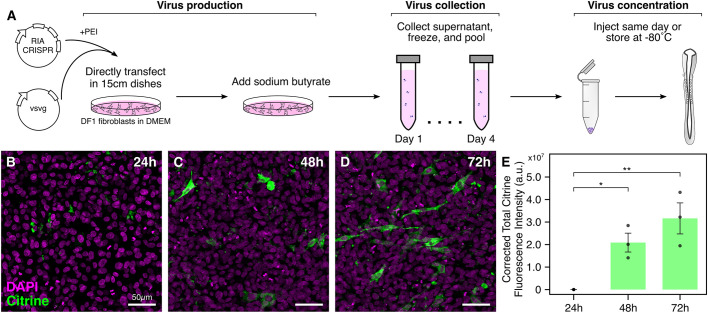
**Incorporating** **the single CRISPR plasmid into RIA retroviruses.** (A) Illustration of optimized protocol to synthesize high-titer avian retroviruses that contain large inserts. Viral particles are produced in 15-cm plates supplemented with sodium butyrate. Supernatant can be pooled and stored at −80°C for several months, and concentrated virus can be injected the same day or stored at −80°C for a few weeks. (B-E) Chicken DF-1 fibroblasts were infected with RIA-CRISPR retrovirus and collected 24 h (B), 48 h (C) and 72 h (D) post-infection. Citrine expression was first observed at the 48 h time point, as validated by the quantification of Citrine CTCF intensity (E; ANOVA followed by Tukey's HSD, **P<0.01, *P<0.05, *n*=3 biological replicates). Error bars represent s.e.m.

Our initial attempt involved injecting two different viruses in the neural tube, one encoding nuclear Cas9 and the other encoding a gene-specific gRNA. However, this approach was unsuccessful in knocking out *Sox10* in the trunk neural crest owing to the low probability of double infection. As an alternative, we explored the possibility of incorporating our single-plasmid design into the RIA virus for targeting later stages of development. However, given the relatively larger size of the *nls-Cas9-nls-2a-Citrine-HH-gRNA^f+e^-HDV* fragment, it was necessary to improve the RIA virus preparation protocol to accommodate this large insert. We achieved this by making three modifications to maintain the functionality of the recombinant virus (Fig. 5A). First, we minimized the time duration that secreted virus stayed in the culture medium by transfecting RIA and VSVG (glycoprotein G from vesicular stomatitis virus) expression constructs directly in 15-cm dishes instead of sequentially passaging transfected cells from 6- to 10- to 15-cm dishes (Chen et al., 1999), which often led to the deletion of inserted genes in the viral particles over time. Second, we used polyethylenimine (PEI), a polymer composed of repeating units of amine group and carbon spacer (Tang et al., 2015), as a less expensive substitute for previously used commercial transfection reagents (Chen et al., 1999), making the transfection process better suited for large-scale production. Finally, we added sodium butyrate, a histone deacetylase inhibitor that has been shown to enhance viral DNA transfection (Sakoda et al., 1999). Together, these modifications allowed us to successfully make high titer retrovirus encoding three large inserts of variable sizes: *nls-Cas9-nls* (4.2 kb), *nls-Cas9-nls-eGFP* (4.9 kb) and *nls-Cas9-nls-2a-Citrine-HH-gRNA^f+e^-HDV* (5.1 kb), hereby referred to as RIA-Cas9, RIA-Cas9-GFP and RIA-CRISPR retroviruses, respectively.

To test the time course of protein expression following infection with the RIA-CRISPR retrovirus, we infected chicken DF-1 fibroblast cells, incubated them at 37°C and 5% CO_2_, and collected cells in triplicate at 24 (Fig. 5B), 48 (Fig. 5C) and 72 h (Fig. 5D) post-infection. Infected cells collected at these three time points were then processed for immunostaining against Cas9 and Citrine. Robust Citrine expression was first observed in cells collected at 48 h (Fig. 5C), suggesting a delay between the time of initial infection and formation of readily detectable protein. Quantification of Citrine CTCF intensity confirmed that significant differences existed in Citrine protein levels between the 24 and 48 h (Fig. 5E; *P*<0.05, ANOVA followed by Tukey's HSD) and 24 and 72 h time points (Fig. 5E; *P*<0.01, ANOVA followed by Tukey's HSD), respectively. These data agree well with our previous findings that detection of robust fluorescent protein *in vivo* in neural crest cells infected with RIA retroviruses is observed after 2 days (Tang et al., 2019). We next sought to test these retroviruses *in ovo* by targeting *Sox10*, *Pax7* and *Pax6* in the neural crest, segmental plate, and retina, respectively.

### Targeting *Sox10* in neural crest derivatives using RIA-CRISPR retroviruses

**Fig. 6.**
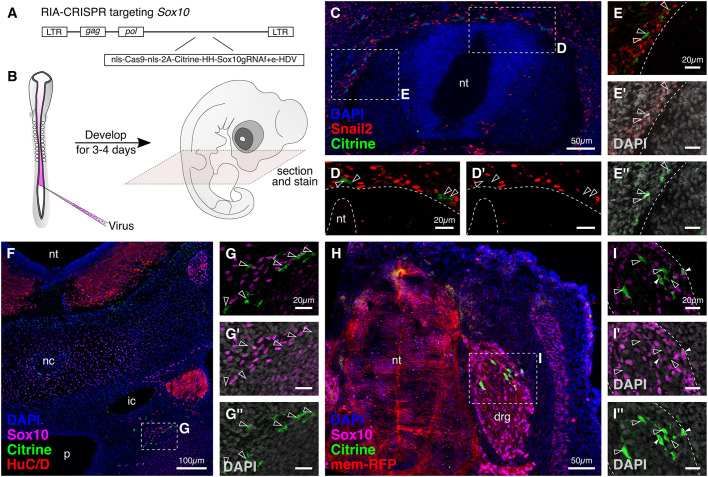
**RIA retrovirus-mediated knockout of *Sox10* in neural crest derivatives.** (A) Schematic for cloning the Sox10-RIA-CRISPR virus plasmid. (B) Sox10-RIA-CRISPR retrovirus was injected into the neural tube lumen at HH10. At HH25, embryos were fixed, cryosectioned and immunostained. (C) Knockout of *Sox10* following emigration from the neural tube does not affect survival but rather causes downregulation of Snail2, a neural crest specification marker. (D-E″) Early (D,D′) and late (E-E″) migrating neural crest cells labeled with Citrine (D′,E″) do not express Snail2 (arrowheads). (F-G″) Transverse section through an injected embryo (F) shows labeled migrating trunk neural crest cells mutant for Sox10 expression close to the sympathetic ganglion (G,G′), with DAPI-labeled nuclei (G″). (H) Labeled cells were also observed in the dorsal root ganglion. (I-I″) Although most Citrine-positive cells were Sox10-negative (unfilled arrowheads), Sox10 protein was detected in a couple of labeled cells (filled arrowheads). drg, dorsal root ganglion; ic, internal carotid; nc, notochord; nt, neural tube; p, pharynx. Dashed line delineates the neural tube in D-E″, and the boundary of the dorsal root ganglion in I-I″.

*Sox10* is known to affect differentiation of several neural crest lineages. Therefore, we cloned the cistronic fragment *nls-Cas9-nls-2A-Citrine-HH-Sox10gRNA^f+e^-HDV* into the RIA viral vector to generate a Sox10-RIA-CRISPR retrovirus (Fig. 6A) that was injected into the lumen of the chick neural tube (Fig. 6B) at HH10 (*n*=20) and examined the subsequent effects on neural crest derivatives at a population level. High-titer RIA-membrane-RFP retrovirus was co-injected as a readout for infection efficiency. As expected, RIA infection failed to robustly label migratory neural crest cells shortly after delamination owing to the time needed to generate proteins after viral integration (Fig. 5E). The proportion of Citrine-expressing cells increased with time, reflecting synthesis of Cas9 protein and gRNA molecules that results in *Sox10* knockout at later stages of neural crest migration. Embryos at >2 days of development contained clusters of Citrine^+^ cells migrating along the ventromedial neural crest migratory pathway (Fig. 6C). Contrasting with early loss of *Sox10*, neural crest cell death was not observed. Rather, there was loss of the neural crest transcription factor Snail2 in migratory neural crest cells (Fig. 6D-E″), and Sox10 protein was diminished in the Citrine-labeled cells (Fig. 6F-G″). That these cells did not undergo premature apoptosis and appeared to migrate properly suggested that *Sox10* plays distinct roles in early and later stages of neural crest development, and confirms that our retroviral approach can be used to analyze phenotypes at later stages.

After 4 days, labeled cells were observed in the dorsal root ganglion in transverse sections through the trunk (Fig. 6H). Immunostaining revealed discrete clusters of Citrine-labeled cells, 96±2.7% of which had lost Sox10 protein (Fig. 6I-I″), suggesting a clean knockdown. As expected given the potential perdurance of protein generated prior to the knockout and/or incomplete knockout in cells in which only a single allele was deleted, a low level of Sox10 protein was detectable in 4±2.7% of the Citrine-labeled cells (Fig. 6I′, white filled arrowheads). Given the relatively sparse Citrine expression, it is easy to identify a small number of mutant cells in an otherwise normal embryonic background. Taken together, these results suggest that our RIA-CRISPR retroviruses can be used to decouple the early function of *Sox10* in maintaining neural crest cell viability from its later role in cell differentiation.

### Targeting *Pax6* in the chick retina

In the chick, outgrowth of the optic vesicles in the forebrain marks the first sign of eye development (O'Rahilly and Meyer, 1959). This is followed by formation of layers with the neural retina as the inner layer and outer retinal pigment epithelium. In *Pax6^−/−^* mice, the optic cup and other ocular tissues fail to develop (Marquardt et al., 2001), suggesting that expression of this paired-box transcription factor plays a crucial role in the survival of optic vesicle cells (Canto-Soler and Adler, 2006). After optic vesicle formation, *Pax6* maintains multipotentiality of retinal progenitor cells (Marquardt et al., 2001). Consistent with this, in *Drosophila* and *Xenopus* embryos, ectopic expression of the *Pax6* coding sequence results in formation of ectopic eyes (Chow et al., 1999; Halder et al., 1995). Although inducible Cre-LoxP has contributed greatly to understanding the role of *Pax6* in murine eye development, the only feasible method for genetic perturbations in the chick retina has been electroporation at different stages (Sebastián-Serrano et al., 2012), which has limitations, including reduced tissue accessibility and lack of permanent labeling of transfected cells.

**Fig. 7.**
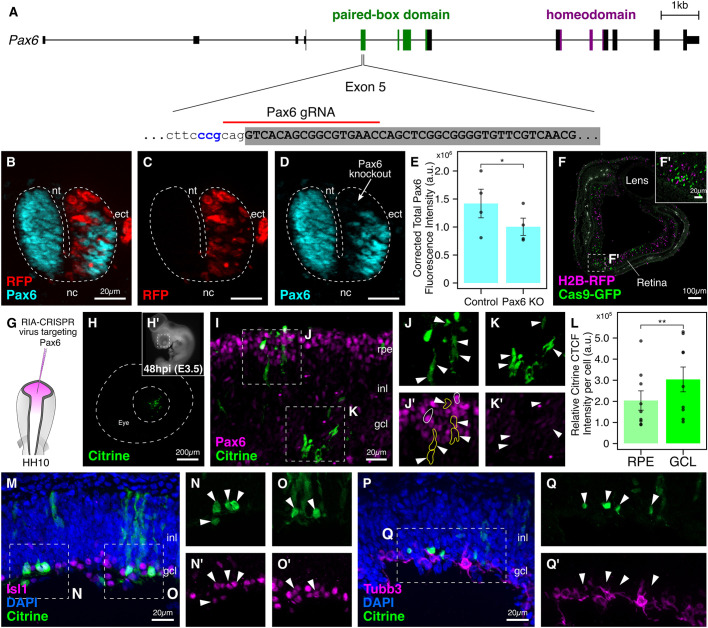
**Targeting *Pax6* in the developing chick retina using RIA-CRISPR retrovirus.** (A) The genomic locus for *Pax6* in the chick with paired-box (green) and homeodomain (purple) indicated. Of gRNAs tested, the one targeting the splice acceptor site of exon 5 was most efficient. (B-E) The right side of gastrula-stage embryos was electroporated with constructs encoding Cas9, RFP and *Pax6* gRNA. Transfected cells (C) lacked expression of Pax6 (D), as seen in the overlay image (B). Dashed line delineates the neural tube. Cross-sections through embryos electroporated with the single CRISPR plasmid targeting *Pax6* were used to quantify fluorescence intensity of Pax6 in the neural tube; results reveal a significant difference in Pax6 expression between the knockout and control sides (E; *P<0.05, paired Student's *t*-test, *n*=4 sections from 2 representative embryos). (F,F′) Section through the eye at embryonic day 4 (F) injected with RIA-Cas9-GFP and RIA-H2B-RFP retroviruses. Labeled cells were evenly distributed (F′) through all layers of the developing retina. (G) The injection strategy for targeting *Pax6* in retinal progenitor cell precursors residing in the optic vesicle. (H,H′) Citrine expression (H) in the developing eye (H′) was first observed 48 h post-infection. Dashed lines delineate the developing eye. (I-K′) Citrine-labeled cells (I,J,K) in both the retinal pigmented epithelium and the ganglion cell layers were negative for Pax6 expression (J′,K′, arrowheads). Intensity of Citrine expression together with endogenous Pax6 levels allowed identification of two clones in the retinal pigmented epithelium layer (J′): outlined in white – high Citrine, high Pax6; outlined in yellow – low Citrine, no Pax6. (L) Relative Citrine CTCF intensity per labeled cell was significantly higher in the ganglion cell layer (GCL) compared with the retinal pigmented epithelium (RPE) (*P<0.01, paired Student's *t*-test, *n*=9). (M-O′) 77.08±5.5% of Citrine-labeled cells (M,N,O) were biased towards an amacrine cell fate based on expression of Islet1 (N′,O′, arrowheads). Several migratory cells were observed in the inner nuclear layer. (P-Q′) 80.24±3.1% of Citrine-labeled cells (P,Q) in the ganglion cell layer failed to turn on Tubb3, a marker for differentiated mature neurons (Q′, arrowheads). ect, ectoderm; gcl, ganglion cell layer; inl, inner nuclear layer; nc, notochord; nt, neural tube; rpe, retinal pigmented epithelium. Error bars represent s.e.m.

To establish the feasibility of applying our modified RIA-CRISPR retroviruses to the chick retina, we targeted *Pax6* in the optic vesicle. We first sought to identify a functional gRNA that would successfully knock out chick *Pax6*. The genomic locus in the chicken genome spans 16.8 kb on chromosome 5 and consists of 14 exons, with the paired-box domain spanning between exons 5 to 8, and the homeodomain spanning between exons 9 to 11 (Fig. 7A). We designed two gRNAs, one targeting the splice acceptor site of exon 5 and another targeting the splice acceptor site of exon 7, and individually tested their efficacy by electroporating the right side of gastrula-stage embryos with constructs encoding Cas9, the two individual gRNAs and nuclear RFP (as a transfection control). Only the gRNA targeting the splice acceptor site of exon 5 was successful in knocking out *Pax6* in electroporated cells within the neural tube (Fig. 7B-D), as confirmed by loss of Pax6 protein (Fig. 7D). Next, we incorporated this protospacer into our single-plasmid design to synthesize the *CAG>nls-Cas9-nls-2A-Citrine-HH-Pax6gRNAf+e-HDV* construct, which we electroporated on the right side of gastrula-stage embryos, which were fixed and processed for cryosectioning and immunostaining at HH9. The quantified Pax6 CTCF intensity (four sections obtained from two representative embryos) was significantly different in the neural tube of the knockout side compared with the control side (Fig. 7E; *P*<0.05, paired Student's *t*-test). Therefore, we selected this gRNA for all our subsequent experiments.

Given the large size of Cas9 and the fluorescent protein insert, we began by confirming that injection of a large-insert virus would sufficiently label cells in the developing chick retina and not interfere with normal retinal development. To this end, we utilized the RIA-Cas9-GFP retrovirus and injected it in the optic vesicle of HH9 chick embryos, co-injecting RIA-H2B-RFP retrovirus as an infection control. Embryos were harvested after 3 days, and the eyes were dissected, cryosectioned and immunostained. Imaging of sections revealed distinct clusters of GFP^+^ and RFP^+^ cells in the developing retina and the retinal pigmented epithelium layer (Fig. 7F). The eye developed normally, and cells labeled with the two retroviruses were evenly distributed throughout the presumptive outer and inner nuclear layers (Fig. 7F′), confirming lack of interference of the large insert size with normal eye development, efficiency of infection, and subsequent fluorescent protein expression. Accordingly, we incorporated the *nls-Cas9-nls-2A-Citrine-HH-Pax6gRNA^f+e^-HDV* fragment into the RIA vector to synthesize Pax6-RIA-CRISPR virus for injection in the optic vesicle of HH9 embryos (Fig. 7G). Following injections, expression of Citrine was first observed in the developing eye at 48 h post-injection (Fig. 7H,H′).

Next, we harvested injected embryos following 4-5 days of development (*n*=25) and obtained transverse sections through the dissected eyes. Given the delay between infection and expression of Citrine, targeting *Pax6* in the optic vesicle allows us to decouple the later from the early roles of Pax6. Indeed, immunostaining of sections through eyes infected with the Pax6-RIA-CRISPR retrovirus demonstrated several distinct clones distributed along the retinal pigmented epithelium, the ganglion cell layer, and as elongated migratory cells in the inner cell layer (Fig. 7I). Using the intensity of Citrine expression and levels of endogenous Pax6 protein, we were able to identify labeled cells within the retinal pigmented epithelium layer that are likely to be sister cells (Fig. 7J,J′). One such ‘clone’ had high levels of Citrine (outlined in white) and retained Pax6 expression, whereas the second had low levels of Citrine (outlined in yellow) and diminished Pax6 expression. Labeled cells with high Citrine (Fig. 7K) and downregulated Pax6 expression (Fig. 7K′) were also scattered within the developing ganglionic cell layer. Interestingly, the signal intensity of Citrine was stronger in the ganglionic cell layer compared with the inner nuclear or the retinal pigmented epithelium layer (*n*=9), with a statistically significant difference in the Citrine CTCF intensity per unit cell between the two layers (Fig. 7L; *P*<0.01, paired Student's *t*-test).

Finally, we assessed effects of the Pax6-RIA-CRISPR retrovirus on the fate of retinal progenitor cells by immunostaining cross-sections through the eyes with antibodies against *Islet1*, a marker for amacrine cells, one of the six cell types that are derived from the retinal progenitor cells. In the chick retina, retinal gangliogenesis begins around embryonic day 6, marked by expression of the *Brn3* family of proteins (Doh et al., 2010; Liu et al., 2000). Previous findings have suggested that temporally restricted loss of Pax6 is associated with preferential generation of amacrine interneurons (Marquardt et al., 2001). Accordingly, examination of representative sections revealed that 77.08±5.5% of Citrine^+^ cells  in the ganglionic cell layer expressed Islet1 (Fig. 7M-O′), supporting the possibility that the virally infected cells initiate amacrine differentiation. We tested whether these labeled cells were terminally differentiated neurons by staining sections for β-III tubulin (Tubb3, also known as Tuj1), a marker for post-mitotic mature neurons. Of the representative sections examined, 80.24±3.1% of Citrine^+^ cells within the ganglionic cell layer were Tubb3-negative, suggesting that these were amacrine precursors rather than prematurely differentiated amacrine neurons (Fig. 7P-Q′). This was consistent with results in a Pax6*^lacZ^* mutant mouse model, where cells failed to turn on Tuj1 (also known as Tubb3) in the absence of Pax6 (Oron-Karni et al., 2008). Of note, the RIA approach successfully labeled a subpopulation of retinal cells, suggesting that this sparse labeling approach is well-suited for examination and long-term tracking of mutant cells in an otherwise normal environment. Taken together, our results show that the RIA-CRISPR viruses can be used for later-stage knockdown of genes of interest in the chick retina, enabling new lines of investigation previously limited to genetically tractable model systems.

### Targeting *Pax7* in the neural crest and presomitic mesoderm

**Fig. 8.**
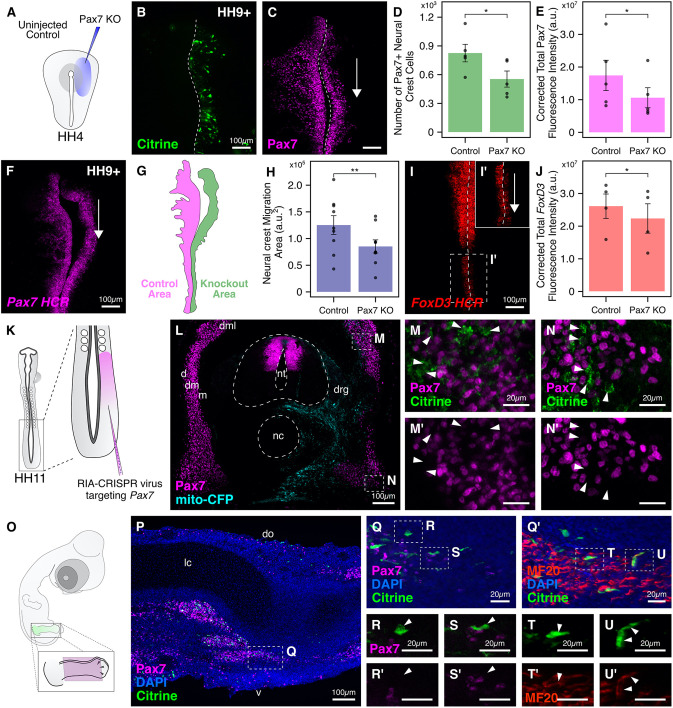
**Targeting *Pax7* in the dermomyotome and hindlimb using RIA-CRISPR retrovirus.** (A) Electroporation strategy for knocking out *Pax7* in the neural crest. The right side of gastrula-stage embryos was electroporated with the single CRISPR plasmid. (B) Embryos developed to HH9+ displayed robust expression of Citrine on the right side. Dashed lines mark the midline. (C-E) The engineered single plasmid efficiently knocked out *Pax7* (C) on the electroporated side. The number of neural crest cells (D; *P<0.05, paired Student's *t*-test, *n*=5) and Pax7 fluorescence intensity (E; *P<0.05, paired Student's *t*-test, *n*=5) were significantly reduced on the knockout compared with control side. (F-H) *In situ* HCR showed that *Pax7* mRNA levels were also downregulated on the knockout side. The arrow reflects downregulation on the right side. The boundary of migration area (G) occupied by the cranial neural crest cells in F was identified using the position of the lateral-most leader cell for both control (magenta) and knockout (green) sides. Quantification of this difference revealed a significant reduction in neural crest migration on the knockout side (H; **P<0.01, paired Student's *t*-test, *n*=9). (I-J) In the absence of *Pax7*, the expression of the neural crest specifier gene *FoxD3* is severely downregulated (I), especially in the hindbrain, where the effect of the CRISPR plasmid is most penetrant (I′). Quantification of the *FoxD3* fluorescence intensity revealed significant difference between knockout and control sides (J; *P<0.05, paired Student's *t*-test, *n*=4). (K) Injection strategy for targeting *Pax7* in the presomitic mesoderm of HH11 embryos using the Pax7-RIA-CRISPR retrovirus. (L) Transverse section through embryos developed to HH25 shows positive labeling with tracer virus (mito-CFP) on the injected side. (M-N′) Citrine^+^ cells in the dorsal (M,M′) and ventral (N,N′) aspects of the dermomyotome were *Pax7*-negative (arrowheads). (O) In infected embryos developed to embryonic day 6, the limb was dissected and sectioned along the coronal plane, shown in magenta. (P) Several Citrine^+^ mutant cells were observed in the dorsal and ventral muscle mass. (Q-U′) *Pax7*-mutant cells (Q,S,S′) robustly turn on the skeletal muscle marker MF20 (Q′,T-U′). Several Citrine^+^/MF20^−^ cells that failed to turn on Pax7 (R,R′, arrowheads) were observed close to ventral and dorsal muscle masses, perhaps corresponding to muscle satellite cell precursors. Q and Q′ are adjacent sections that captured the same cell. do, dorsal; d, dermatome; dm, dermomyotome; dml, dorsomedial lip; drg, dorsal root ganglion; lc, limb cartilage; m, myotome; nc, notochord; nt, neural tube; v, ventral. Error bars represent s.e.m.

In early chick embryos, neural crest cells are induced shortly after gastrulation at the neural plate border, a region between the neural plate and non-neural ectoderm (Gandhi and Bronner, 2018). Expression of *Pax7* primes neural plate border cells to become neural crest at the expense of other lineages (Gandhi et al., 2020b; Roellig et al., 2017). To test that the single-plasmid strategy reduces Pax7 levels, we electroporated the right side of gastrula-stage embryos with the *CAG>nls-Cas9-nls-2A-Citrine-HH-Pax7gRNA^f+e^-HDV* plasmid (Fig. 8A), which contained a previously validated *Pax7* protospacer (Gandhi et al., 2017). As an internal control, the left side was either not injected, or injected with a control plasmid (*CAG>nls-Cas9-nls-2A-Citrine-HH-CtrlgRNA^f+e^-HDV*), with similar results in both cases. Embryos were cultured *ex ovo* until HH9+ (Fig. 8B), then fixed and stained for Pax7 expression. Levels of Pax7 were notably reduced in cranial neural crest cells (Fig. 8C), as quantified by measuring the number of cranial neural crest cells (*n*=5) and Pax7 CTCF intensity (*n*=5), on the treated and control sides. Following *Pax7* knockout, we observed a 33% reduction in the number of neural crest cells (Fig. 8D; *P*<0.05, paired Student's *t*-test) and a 37% reduction in the Pax7-CTCF intensity (Fig. 8E; *P*<0.05, paired Student's *t*-test). Furthermore, levels of *Pax7* mRNA were also notably downregulated, as demonstrated by *in situ* HCR (Fig. 8F). Next, we focused on the area occupied by neural crest cells on the control and knockout sides. *Pax7* mRNA signal was used to trace the extent of migration on either side of the embryo (Fig. 8G). Indeed, the migration area (*n*=9) was significantly downregulated by 31%, with neural crest cells occupying an area of 12,5143±17,353 a.u.^2^ on the control side compared with 85,079±12,109 a.u.^2^ on the treatment side (Fig. 8H; *P*<0.01, paired Student's *t*-test). Finally, given that *Pax7* acts upstream of the neural crest specifier gene *FoxD3* and directly regulates its activity by binding to a proximal enhancer (Simões-Costa et al., 2012), we processed knockout embryos for *in situ* HCR against *FoxD3* (Fig. 8I,I′), and noticed a significant reduction in its CTCF intensity (*n*=4) on the right side of the embryo (Fig. 8J; *P*<0.05, Student's *t*-test), particularly in the hindbrain (Fig. 8I′).

As the embryo develops, *Pax7* is expressed in other tissues, including the dermis and skeletal muscle progenitors that reside in the dorsal dermatome and ventral myotome. The myogenic progenitors delaminate from the myotome, migrate to the developing limb bud, and proliferate (Buckingham et al., 2003). Once these cells are in the limb bud, expression of *MyoD*, a determination factor for the myogenic lineage, results in their differentiation into skeletal muscle (Rudnicki et al., 1993). Expression of *Pax7* is crucial for the proper development of muscle satellite cells, which are also derived from the *Pax7*-expressing precursors residing in the dermomyotome (Von Maltzahn et al., 2013). To diversify the application of our single-plasmid approach, we sought to target *Pax7* not only in presumptive neural crest, but also later in the muscle progenitor population of the developing dermomyotome.

We cloned this transgene into the RIA viral vector and generated RIA-CRISPR virus with the aim of infecting cells that express *Pax7* in the dorsolateral region of the developing somites that gives rise to the dermis and skeletal muscles. Although *in ovo* electroporation of the presomitic mesoderm is possible (Itasaki et al., 1999), the technique requires puncturing the egg yolk to insert the positive electrode, which can reduce viability. Instead, we injected the *Pax7* RIA-CRISPR virus directly into the segmental plate of HH11 chick embryos on the right side (Fig. 8K) and allowed them to develop for 3-4 days post-infection (*n*=18). RIA-CFP fluorescent protein tagged with a mitochondrial localization sequence served as an infection control. At HH25, embryos were harvested, sectioned through the region caudal to the injection site, and immunostained. We found Citrine^+^ infected cells that lacked Pax7 expression on the injected side (Fig. 8L), within the dorsal (Fig. 8M,M′) and ventral (Fig. 8N,N′) aspects of the dermomyotome. The high proportion of infected cells were likely derived from proliferating progenitors that had successful integration of Cas9, Pax7 gRNA and Citrine in their genome. The injected embryos had a high survival rate (>90%), suggesting that targeted retroviral injection was less likely than *in ovo* electroporation to affect long-term viability of embryos.

Finally, in a subset of embryos that were injected with the RIA-CRISPR virus targeting *Pax7* (*n*=6), the hindlimb on the injected side was strongly labeled with Citrine. Therefore, we dissected these hindlimbs (Fig. 8O), made coronal sections, and stained them for the myogenic marker MF20. We found several clusters of Citrine^+^ cells in both the ventral and dorsal muscle mass of the hindlimb (Fig. 8P). To confirm that the cells that successfully differentiated into muscles were indeed mutant for *Pax7* expression, we also stained adjacent sections for Pax7. Consistent with the loss of Pax7 expression in the dermomyotome (Fig. 8L), Citrine^+^ cells did not express Pax7 (Fig. 8Q,S,S′) showing that *Pax7* was successfully knocked out in their progenitors but mutant cells expressed strong levels of MF20 (Fig. 8Q′,T-U′). By contrast, we observed several infected cells that were anatomically adjacent to the muscle cells but were negative for MF20 and Pax7 expression (Fig. 8R), which we speculate correspond to muscle satellite cell precursors (Fig. 8R′), although further characterization is required to validate their identity. Taken together, these results establish an additional potential application of our engineered RIA-CRISPR retroviruses to a tissue that that was previously difficult to access in chicken embryos.

### Coupling RIA-CRISPR retrovirus-mediated perturbation with clonal lineage tracing

**Fig. 9.**
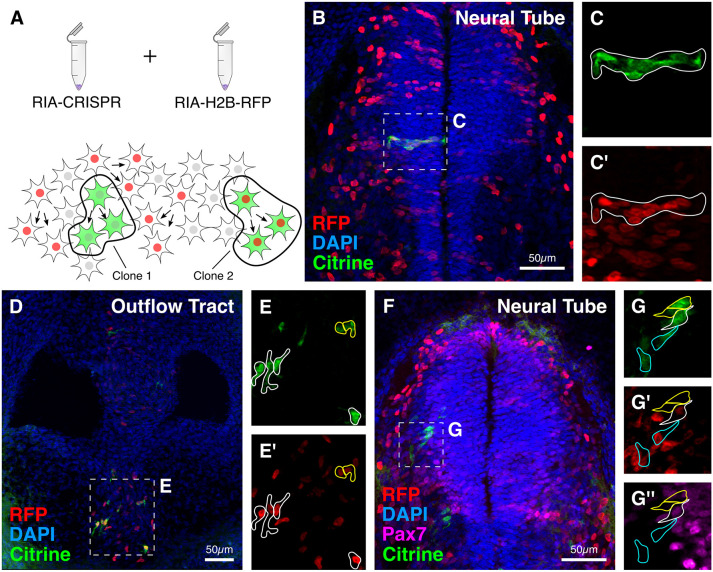
**Proof-of-principle experiment to show application of RIA-CRISPR retroviruses for clonal analysis.** (A) Experimental design for performing clonal analysis using RIA-CRISPR retroviruses. Clones were identified as single-infected cells with shared fluorescence intensity for Citrine, or double-infected cells with shared fluorescence intensity for Citrine and RFP. (B-E′) Examples of clonally related cells observed in the medial neural tube (B) and developing outflow tract (D). In the neural tube, clones (outlined) were horizontally distributed (C,C′), whereas in the outflow tract, clones were distributed evenly within the aorticopulmonary septum (E,E′). (F) In a representative embryo, double labeling with Citrine and RFP, together with intensity of Citrine and RFP and expression levels of Pax7, indicate clonal relationships. (G-G″) This allowed identification of three distinct clones: outlined in white – high Citrine (G), low RFP (G′), low Pax7 (G″); outlined in cyan – low Citrine (G), high RFP (G′), no Pax7 (G″); outlined in yellow – high Citrine (G), no RFP (G′), no Pax7 (G″).

Finally, we investigated whether the RIA-CRISPR retrovirus-mediated gene editing can be used in combination with other RIA viruses for *in vivo* clonal analysis. To establish proof of principle for this application, we chose the developing spinal cord as our tissue of interest, and co-injected RIA-CRISPR retrovirus targeting Pax7 (Citrine^+^) with an RIA retrovirus encoding only nuclear RFP (H2B-RFP) at a volumetric ratio of 10:1 into the lumen of the neural tube of HH12 embryos (Fig. 9A). Following a 3-day incubation *in ovo*, embryos were fixed, cryosectioned and processed for immunostaining, allowing us to identify labeled cells both within the neural tube and in neural crest-derived cells in the periphery.

Building upon our previously published work (Tang et al., 2019), we estimated that the probability of infection with the RIA-CRISPR virus alone was 1:10,000, whereas the probability of double infection with the RIA-CRISPR and RIA-H2B-RFP retrovirus was 1:100,000 (detailed in Materials and Methods). Moreover, given that the number and location of integrations varies with each infection event, the progeny of the infected cell share similar levels of fluorescent protein expression, thus facilitating identification of clonally related cells (Fig. 9A). The labeling in the neural tube was sparse; therefore, we posited that Citrine^+^ cells in close proximity and with similar fluorescence intensity, reflecting the integration site, were likely to be clonally related. In rare cases, we also observed cells that were double labeled with RIA-CRISPR and RIA-H2B-RFP retroviruses, and we predicted that these cells also represented a clone. As expected, if *Pax7* was successfully knocked out in a particular cell, the entire clone also lacked Pax7 expression. If only one allele was targeted, then similar levels of residual Pax7 expression would be apparent in all clonally related progeny.

Based on these criteria, we identified putative clonally related cells in both the neural tube (Fig. 9B-C′) and periphery (Fig. 9D-E′). Within the developing spinal cord, immunostaining revealed either no or low levels of Pax7 expression in Citrine-labeled cells (Fig. 9G″). The deletion of only one of the alleles in some cases likely accounts for persistence of Pax7 expression in some cells. Interestingly, two different double-labeled clones within the neural tube were distinguishable based on their levels of RFP, Citrine and endogenous Pax7 protein expression (Fig. 9F). One clone of neural tube cells with high Citrine and low RFP (outlined in white) had low Pax7 expression (Fig. 9G-G″), whereas the other adjacent clone with high RFP but low Citrine (outlined in blue) appeared to lack Pax7 expression. Together, we identified 18 double-labeled clones across six embryos, of which 11 lacked Pax7 expression. In addition, Citrine^+^ only clones were identified based on sparse labeling and linear distribution along the coronal plane within the neural tube (*n*=22 Citrine^+^ only clones), of which ten lacked Pax7 expression; one such example is outlined in yellow (Fig. 9G-G″). Together, these results demonstrate that long-term lineage analysis can be accomplished with our RIA-CRISPR retroviruses in chick embryos at either the single-cell or population level. In principle, our single construct can be incorporated into a number of different viruses, including lentiviral or adenoviral vectors, and can therefore be utilized to assess gene functions in diverse morphogenic events within an otherwise normal embryonic environment in multiple species.

## Conclusions

Cell lineage analysis is an essential tool for following cell fate by marking progenitor cells and determining the cell types into which they differentiate. By combining lineage tracing with perturbation methodologies, one can challenge the cell's developmental potential and interrogate the role of particular genes in normal developmental processes. Our single-plasmid technology enables concomitant delivery of guide RNAs, Cas9 and a fluorescent tag into embryonic cells by electroporation or via replication-incompetent avian retroviral delivery. By targeting genes in the neural tube and neural crest, retina, and segmental plate, we have demonstrated the versatility of our engineered RIA-CRISPR viruses. This single-plasmid approach could also be incorporated in other virus families, including adeno- and lenti-viruses for application in other species. Moreover, we have expanded the application of the CRISPR-Cas9 system to investigations involving live imaging. These modified constructs can be efficiently adapted to several tissues in the developing embryo to study behavior of mutant cells for either short-term or long-term analysis in their endogenous wild-type environment.

## MATERIALS AND METHODS

### Chicken embryos

Fertilized chicken embryos (*Gallus gallus*) were commercially obtained from local farms (Sun Valley and AA Laboratory Eggs, CA, USA) and developed to desired stages as per Hamburger and Hamilton (1951) in humidified incubators set to 37°C. For *ex ovo* experiments, embryos were developed for 18-21 h, whereas for *in ovo* experiments, eggs were incubated for 36-48 h to obtain HH10-HH12 embryos. *Ex ovo* electroporations were performed as previously described (Sauka-Spengler and Barembaum, 2008) by passing five 5.2 V pulses for 50 ms each every 100 ms. The electroporated embryos were then cultured in 1 ml albumin supplemented with penicillin/streptomycin (PenStrep) at 37°C. The next day, the embryos were screened for transfection efficiency (high fluorescence intensity within the neural tube in whole-mount embryos), and unhealthy and/or poorly transfected embryos were discarded. All plasmids were injected at a final concentration of 2.5 µg/µl. Specific-pathogen-free fertilized chicken eggs were also commercially obtained from Charles River Laboratories and incubated for similar durations as above.

### Molecular cloning

The plasmid *CAGG>nls-Cas9-nls-2a-Citrine* (Addgene plasmid #92393, deposited by Tatjana Sauka-Spengler) was modified by inserting an EcoRI restriction enzyme site downstream of the Citrine sequence. The AscI-nls-hCas9-nls-2a-ClaI-Citrine-EcoRI/NotI fragment (using Cas9 from Gandhi et al., 2017) was ordered as a gene block from Twist Biosciences to generate the ‘Single-CRISPR' vector (Addgene plasmid #169097). The HH and HDV ribozyme sequences were PCR amplified from the plasmid pUDP044 (Addgene plasmid #101168, deposited by Jean-Marc Daran). A modified ‘shuttle vector' (Addgene plasmid #169098) was generated by inserting two BsaI restriction enzyme sites on opposite strands between the HH and HDV ribozyme sequences. Ctnnb1, Pax7, Sox10, Pax6 and the control protospacers were ligated into the shuttle vector, which allowed the amplification of the HH-gRNAf+e-HDV fragment. These fragments were directionally cloned downstream of Citrine in the modified CAGG>nls-Cas9-nls-2a-Citrine vector [for ligation into the RIA retrovirus backbone (Tang et al., 2019) under the regulation of the RIA promoter] or the ‘single-CRISPR' vector (for chick embryo electroporations). See supplementary Materials and Methods for a detailed protocol. gRNA protospacers used in this study are shown in Table S1.

### Tissue culture transfection experiments

Human osteosarcoma cells (U2OS, ATCC CRL-3455) were cultured in McCoy's 5a Modified Media (Thermo Fisher), and chicken fibroblasts (DF-1, ATCC CRL-12203) were cultured in DMEM (Corning), both supplemented with 10% fetal bovine serum (Gibco) and PenStrep (Corning) at 37°C with 5% CO_2_. Cells were seeded at a density of 0.04×10^6^ cells per well into ibidi µ-Slide 8-well glass bottom imaging slides (ibidi, 80827) and incubated for 48 h before transfection using Lipofectamine 3000 (Invitrogen).

### Virus preparation and injection

Virus particles were harvested and concentrated using a protocol optimized for large insert size (see supplementary Materials and Methods). Viruses that contained fluorescent reporters were synthesized using previously described protocols (Li et al., 2019). For the optimizations, chick DF-1 fibroblasts cells were transfected with the *RIA-nls-Cas9-nls-2a-Citrine-HH-gRNA-HDV* plasmid together with a plasmid encoding VSVG mixed with PEI. The day after transfection, sodium butyrate was added to the media. Six hours later, the media was replaced with fresh DMEM and the cells were given 24 h to start producing virus particles. The supernatant was collected every 12 h for several days and stored at −80°C to enable pooling. Finally, the viruses were concentrated by centrifugation at 75,500 ***g*** for 1.5 h and the pellet dissolved in DMEM on the day of the experiment. To enable visualization of the injection solution, 0.5 µl of 2% red food dye was added to the virus. *RIA-nls-Cas9-nls-2a-Cit-HH-Sox10.gRNA^f+e^-HDV* and *RIA-nls-Cas9-nls-2a-Cit-HH-Pax6.gRNA^f+e^-HDV* viruses were injected *in ovo* into the lumen of the neural tube of HH10 embryos, whereas the *RIA-nls-Cas9-nls-2a-Cit-HH-Pax7.gRNA^f+e^-HDV* virus was injected in the segmental plate of HH11 embryos. The embryos were developed for 3-5 days following injections, harvested, screened for the expression of Citrine, and processed for cryosectioning and immunostaining.

### Clonal analysis in the neural tube with RIA-CRISPR viruses

Pax7-RIA-CRISPR and RIA-H2B-RFP retrovirus were mixed at a volumetric ratio of 10:1 and injected into the lumen of the neural tube in chick embryos at HH12. Then, 72 h post injection, embryos were fixed with 4% paraformaldehyde (PFA) in PBS for 30 min at 4°C. To perform clonal analysis, the trunk neural tubes were dissected and embedded in gelatin. Transverse cryosections were made across the spinal cord to reveal clonal structures. Subsequently, frozen sections were washed in 1× PBS at 42°C until residual gelatin was removed. Immunohistochemistry was performed using primary antibodies against Citrine, RFP and Pax7. Slides were counterstained with DAPI to reveal tissue morphology and imaged on a Zeiss Imager M2 with an ApoTome module. All images were processed using Apotome Raw Convert in Zen Blue software. Single infection with the RIA-CRISPR virus and double infection with RIA-CRISPR and RIA-H2B-RFP viruses were both defined as rare clones. Cells distributed horizontally along the neural tube, together with those that shared similar fluorescence intensity for Citrine, RFP and/or endogenous Pax7 were identified as clonally related. Because clones were generally thicker along the anterior-posterior axis than the transverse sections made, cells expressing the same combination of viruses but spanning multiple transverse sections were recorded as a single clone.

### Calculating the probability of co-infection

The probability of ‘*k*’ number of infections given a specific multiplicity of infection (MOI) ‘λ’ for each virus can be modeled by a Poisson distribution, such that:(1)
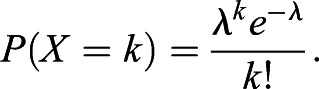


Typically, the titer of RIA retrovirus prepared using our standard protocol is approximately 10^7^ pfu/ml for fluorescent proteins, including the H2B-RFP-encoding retrovirus used in this experiment. Because the RIA-H2B-RFP was diluted at a volumetric ratio of 1:10, the resulting titer of the injected virus was 10^6^ pfu/ml. In our previous work in which we performed clonal analysis on the trunk neural crest (Tang et al., 2019), we had calculated the volume of virus injected in the embryo to be 0.5 µl, which corresponded to 500 retrovirus particles encoding H2B-RFP. Given that each infection event is independent, using the Poisson distribution, the probability of a neural tube cell getting infected with the RIA-H2B-RFP retrovirus is calculated to be:(2)
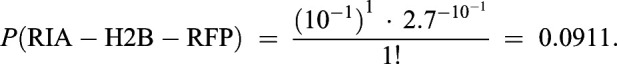


However, the increased insert length for the RIA-CRISPR virus results in a titer that is to 0.1-1% of that of the RIA-H2B-RFP retrovirus. Therefore, the probability of a neural tube cell getting infected with the RIA-CRISPR retrovirus can be calculated as:(3)
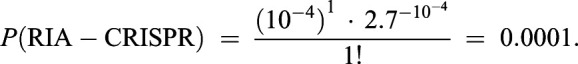
Finally, the probability of co-infection of a single neural tube cell with both the RIA-H2B-RFP and RIA-CRISPR retroviruses can be calculated as:(4)
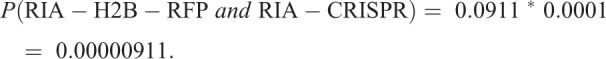


### Slice culture and live imaging

Embryos electroporated with *pCAG>nls-Cas9-nls-2a-Cit-HH-Sox10.gRNA^f+e^-HDV, pCAG>H2B-RFP* and *FoxD3-NC2>Cerulean* plasmids were incubated at 37°C, harvested after 24 h, and screened for the expression of nuclear RFP, which was co-electroporated as a transfection control. Embryos that were poorly transfected were discarded, and the rest were processed as follows: transverse cuts spanning two somites were made through the trunk region using a micro-knife (Fine Science Tools). The slices were washed in Ringer's solution and transferred into a fluorodish containing pre-warmed Neuro-basal media (Gibco) supplemented with glutamine and PenStrep. The slices were positioned under custom-made nylon grids and given 15 min to stabilize, after which the whole fluorodish was transferred into the incubation chamber of a Zeiss LSM 800 microscope for time-lapse imaging. Control embryos were processed in the same way as the mutant embryos.

For imaging the tissue slices, one-photon laser excitation was used at wavelengths of 405, 488 and 561 nm. For these experiments, the 20×/0.8 NA M27 objective was used and *z*-stacks comprising 25 slices spanning 38.4 µm were collected every 10 min for 11 h. The images were imported into Imaris to generate movies.

### *In situ* hybridization, immunostaining hybridization chain reaction

Chromogenic *in situ* hybridization for *Sox10* was performed as previously described (Simoes-Costa and Bronner, 2016; Simões-Costa et al., 2015).

For antibody staining of whole-mount embryos, embryos fixed in 4% PFA were washed in 0.1% Triton X-100 in PBS (PBS-Triton), blocked in 10% donkey serum in 0.5% PBS-Triton for 2 h at room temperature, and incubated in primary antibody solution at 4°C for two nights. On the third day, the embryos were washed in 0.5% PBS-Triton (30 min per wash, six washes) and incubated in secondary antibody solution for two nights at 4°C. Stained embryos were then washed in 0.5% PBS-Triton (30 min per wash, six washes) and processed for imaging and/or cryosectioning.

For antibody staining of cross-sections, slides were degelatinized in 1× PBS for 10 min at 42°C, permeabilized in 10% donkey or goat serum in 0.3% PBS-Triton, incubated in primary antibody solution overnight at 4°C, washed the following day in 1× PBS (four washes, 15 min per wash), incubated in secondary antibody solution at room temperature for 1 h, washed in 1× PBS twice, soaked in 1× PBS containing 0.1 µg/ml DAPI for 2 min, and washed in 1× PBS followed by distilled water. Fluoromount medium was used to mount coverslips on slides.

For antibody staining of cultured cells, cells were washed 24 h post transfection with 1× PBS, then fixed by dropwise addition of ice-cold 100% methanol. Fixed cells were then washed twice with TBST+Ca^2+^ (50 mM Tris+150 mM NaCl+1 mM CaCl_2_+0.1% Tween-20), blocked in 10% donkey serum in TBST+Ca^2+^, then incubated in primary antibody solution overnight at 4°C. The following day, cells were washed three times with TBST+Ca^2+^ then incubated in secondary antibody solution at room temperature for 1 h, washed three times with TBST+Ca^+2^, and imaged using a Zeiss LSM 800 inverted confocal microscope.

Primary antibodies used in this study were: mouse anti-Cas9 (Diagenode, C15200216; 1:500), goat anti-GFP (Rockland Immunochemicals, 600-101-215; 1:500), rabbit anti-RFP (MBL, PM005; 1:500) rabbit anti-GFP (Abcam, ab290; 1:500), rabbit anti-Pax6 (BioLegend, PRB-278P; 1:250), mouse IgG2b anti-HuC/D (Invitrogen, A21271; 1:500), mouse IgG2a anti-Tubb3 (BioLegend, 801201; 1:500), goat anti-Sox10 (R&D Systems, AF2864; 1:200), mouse IgG1 anti-Pax7 (Developmental Studies Hybridoma Bank; 1:10), mouse IgM anti-HNK1 (Developmental Studies Hybridoma Bank; 1:5), mouse IgG2b anti-Islet1 (Developmental Studies Hybridoma Bank; 1:100), rabbit anti-Snail2 (Cell Signaling, 95855; 1:200), mouse IgG2b anti-MF20 (Developmental Studies Hybridoma Bank; 1:100), mouse IgG1 anti-β-catenin (Abcam, ab6301; 1:250). Primary antibodies were detected using Alexa Fluor 350/488/568/633/647-conjugated secondary antibodies (Molecular Probes, A11055, A21240, A11034, A21245, A21146, A21136, A21082 and A21238; 1:250).

For HCR, the manufacturer's (Molecular Technologies) recommended protocol was used as previously described (Gandhi et al., 2020b).

### Cryosectioning and imaging

Whole-mount embryos were fixed in 4% PFA at 4°C for 20 min, which was followed by three washes in 1× PBS at room temperature. Fixed embryos were incubated in 5% and 15% sucrose at 4°C for one night each. The following day, embryos were transferred to molten gelatin, incubated at 37°C for one night, embedded in silicone molds, frozen in liquid nitrogen, and stored at −80°C. Embedded embryos were sectioned on a micron HM550 cryostat to obtain 16 µm or 20 µm sections. Embryos and sections were imaged on a Zeiss Imager M2 with an ApoTome module. Images were post-processed in FIJI imaging software (Schindelin et al., 2012). For corrected total cell fluorescence (CTCF) calculations, the following formula was used:

CTCF=integrated density – (selected area×mean background fluorescence).

To count the number of Citrine^+^ in individual layers of the chick retina, a median filter was applied to 8-bit images, followed by a Bernsen-based auto local-thresholding method (Bernsen, 1986) and watershed segmentation to identify cell boundaries. To count the number of cells, the ‘Analyze particles’ function was used.

### Statistics

For direct comparisons between two conditions, a one-sided pairwise Student's *t*-test was performed to calculate significance after the conditions for normality were verified. For multiple comparisons, ANOVA followed by Tukey's HSD correction was employed. Experiments were repeated on different days to ensure uniformity. No data were excluded. Group allocation was random and different batches of fertilized eggs were separately incubated in different incubators and electroporated with fresh DNA solutions.

## Supplementary Material

Reviewer comments
