# Correction: A single-plasmid approach for genome editing coupled with long-term lineage analysis in chick embryos

**DOI:** 10.1242/dev.201078

**Published:** 2022-07-11

**Authors:** Shashank Gandhi, Yuwei Li, Weiyi Tang, Jens B. Christensen, Hugo A. Urrutia, Felipe M. Vieceli, Michael L. Piacentino, Marianne E. Bronner

There was an error in Development (2021) **148**, dev193565 (doi:10.1242/dev.193565).

In the supplementary Materials and Methods, the HDV-NotI-Rev primer was incorrect.

**Corrected:**

HDV-NotI-Rev: 5′ - aaagcggccgcGTCCCATTCGCCATGCCGAA - 3′

**Original:**

HDV-NotI-Rev: 5′ - aaaGAATTCtgggagCTGATGAGTCCGTGAGGACG - 3′

The PDF version of the supplementary information has been corrected.

The authors apologise to the readers for these errors and any inconvenience they may have caused.

