# Supplementary material for: A single-plasmid approach for genome editing coupled with long-term lineage analysis in chick embryos
Source: Development. 2021 Apr 15;148(7):dev193565. doi: 10.1242/dev.193565 (PMC8077534; doi:10.1242/dev.193565)
Supplement: Reviewer comments [file develop-148-193565_review_history.pdf]

A single-plasmid approach for genome editing coupled with long-term lineage analysis in chick embryos

Shashank Gandhi, Yuwei Li, Weiyi Tang, Jens B. Christensen, Hugo A. Urrutia, Felipe M. Vieceli, Michael L. Piacentino and Marianne E. Bronner

DOI: 10.1242/dev.193565

Editor: James Briscoe

Review timeline

Original submission:	3 June 2020
Editorial decision:	3 July 2020
First revision received:	14 January 2021
Editorial decision:	4 February 2021
Second revision received:	9 February 2021
Accepted:	23 February 2021

Original submission

First decision letter

MS ID#: DEVELOP/2020/193565

MS TITLE: A combinatorial approach for genome editing and lineage tracing in chick embryos using replication-incompetent avian retroviruses

AUTHORS: Shashank Gandhi, Yuwei Li, Jens Bager Christensen, Weiyi Tang, Hugo A. Urrutia, Felipe M. Vieceli, and Marianne Bronner

I have now received all the referees' reports on the above manuscript, and have reached a decision. The referees' comments are appended below, or you can access them online: please go to BenchPress and click on the 'Manuscripts with Decisions' queue in the Author Area.

As you will see, the referees express considerable interest in your work, but have some significant criticisms and recommend a substantial revision of your manuscript before we can consider publication. The referees request several clarifications and quantifications of the data you report. Overall, these seem reasonable and addressing these questions will strengthen the study. Both Referee 2 and 3 raise the question of whether genomic lesions can be documented. I appreciate that this would be very difficult to do. However, it seems reasonable to discuss the issue and make the caveats clear.

If you are able to revise the manuscript along the lines suggested, which may involve further experiments, I will be happy receive a revised version of the manuscript. Your revised paper will be re-reviewed by one or more of the original referees, and acceptance of your manuscript will depend on your addressing satisfactorily the reviewers' major concerns. Please also note that Development will normally permit only one round of major revision.

We are aware that you may currently be unable to access the lab to undertake experimental revisions. If it would be helpful, we encourage you to contact us to discuss your revision in greater detail. Please send us a point-by-point response indicating where you are able to address concerns raised (either experimentally or by changes to the text) and where you will not be able to do so

© 2021. Published by The Company of Biologists under the terms of the Creative Commons Attribution License (https://creativecommons.org/licenses/by/4.0/).

within the normal timeframe of a revision. We will then provide further guidance. Please also note that we are happy to extend revision timeframes as necessary.

Please attend to all of the reviewers' comments and ensure that you clearly highlight all changes made in the revised manuscript. Please avoid using 'Tracked changes' in Word files as these are lost in PDF conversion. I should be grateful if you would also provide a point-by-point response detailing how you have dealt with the points raised by the reviewers in the 'Response to Reviewers' box. If you do not agree with any of their criticisms or suggestions please explain clearly why this is so.

Reviewer 1

Advance summary and potential significance to field

In this manuscript, Gandhi et al. report a method by which CRISPR gene editing can be paired with lineage tracing studies in Chicken embryos, allowing for long term tracing of mutant cells. The authors present a modified version of previously published CRISPR systems to allow for viral packaging within replication incompetent avian virus enabling long term labelling of edited cells. The authors then demonstrate the activity of the newly generated construct across multiple tissue types using three different guide RNAs. The development of this technique makes a significant contribution to the field of developmental biology by enabling the union and versatility of CRISPR gene editing with lineage tracing, a method that can both be used for live imaging over short developmental periods and mutant cell tracing over longer developmental time windows within chicken embryos. As such this work, will allow for robust genetic interrogation within a historic model organismal system that is amenable to long term live imaging facilitating mechanistic interrogation of many stages in developmental time. However, to fully capitalize on the potential of the reported tools, significant quantification must be added to the figures and text to provide support for the statements made and ensure validity behind the claim of robustness in labelling and genome editing.

Comments for the author

Essential Revisions:

Throughout the manuscript many of the claims made are based on a single image shown in the figure and need quantification
- Fig1E-F – quantify number of co-infected cells compared to total to claim significant reduction. Additonally, no mention of number of embryos compared, need n values
- Fig 2 – quantification quantification of insitus and of cell numbers and embryo numbers through out figure
- Fig 3 - "As expected, Pax6 expression was missing in most of the Citrine+ cells [Figure 3H]," needs quantification.
- Fig 4H,I,J – the text often states "mostly" in reference to either and increase or decrease of fluorescence. This should be quantified. Number of embryos examined needs to be reported.
- Fig 5B-D - quantification of reduction in expression needed to claim significance

Minor comments:
In the text the authors claim, "Together, these results confirm that the Sox10 gRNA molecules were successfully synthesized in transfected cells, and that cranial neural crest migration is inhibited in the absence of Sox10". This claim would benefit from a co-stain for the SOX10 protein and presence of Cas9 showing both lack of endpoint movement coordinated with loss of expression of SOX10. Currently, the broad in situs are not convincing to this end. Additionally, move this conclusion phrase to after the live imaging data is presented.

- In Fig 2G-P what does the dotted line refer to? Include the timesteps in the figure legend. Additionally, why is there no RFP displayed in panels G-K when the text reports that the control embryos had RFP-H2B expression?

© 2021. Published by The Company of Biologists under the terms of the Creative Commons Attribution License (https://creativecommons.org/licenses/by/4.0/).

- In Fig 2G-P, because of the separate frames needed to display the time-lapse, it would be beneficial to show labelled cell tracks to better visualize and quantify the claims made about migration.

- what HH stage are the embryos in Fig 3?

- The authors reference a delay in Citrine mRNA expression which correlates with a delay in KO, a quantification of this delay would be beneficial.

- Because the guides used for SOX10 and PAX7 have already been developed, it is confusing when the details on the sequence and binding location of the PAX6 guide are highlighted in the text and figures. The authors should make it very clear in the main text that the other guides are taken from previously published studies.

- Line 406 of the main text switches from terming embryos in HH stages to E4.5. which is confusing for the reader. Please provide consistent or expanded explanation.

Reviewer 2

Advance summary and potential significance to field

Although the title of this manuscript and the abstract promise a method for lineage analysis based on replication-incompetent retroviral delivery of CRISPR-Cas9 constructs, the bulk of this paper is focused on generating presumed knock-outs using this technique. From this point of view the advance is relatively minor, since several other groups around the world have already shown convincing loss of function using CRISPR-Cas9 in avian embryos (chick and quail) targeting various genes both to somatic and germ line cells, in some cases also using various viral vectors. Because there is great need in the field for this, I was hoping that the combination of RCAS as a replication incompetent vector, with the opportunity to express different combinations of labels (perhaps made more diverse using rare recombination events, or scarring, or combinatorial events) giving rise to multiple tags allowing entire lineage trees to be followed. Unfortunately despite the focus of the abstract and introduction and to some extent the title, this paper does not contain this.

Comments for the author

For a paper that focuses so strongly on gene knockout, in my opinion it is essential to show that such a knockout has actually occurred. In this case, the only evidence provided that target genes like Sox10, Pax6 and Pax7 have indeed been edited out of the genome is the apparent absence of fluorescence using antibody staining. There could be several possible reasons for lack of expression including effects on transcription and/or translation or other issues, and to make strong definitive statements like "... demonstrating that the Sox10 gene and protein were deleted" (p 10, lines 257-259), or more problematically "... we observed several citrine-positive MUTANT cells distributed around the retina, suggesting that Pax6 was NO LONGER REQUIRED for the survival of these cells." (p. 12, lines 331-332) really requires direct demonstration of the editing event, at least using genomic PCR with specific primers, or better by sequencing targeted cells (although this may be difficult given the mosaic nature of the transfection and the likely multiple/diverse targeting events). But I think this is really important for a paper like this to reach the conclusions presented - this is even more important because there are examples in the paper where the results are not quite what is expected based on this interpretation, for example "a couple of cells" in some cases that clearly have been targeted but do have expression of the supposedly knocked-out gene. Presumably in a finite number of cases only one of the alleles will have been deleted, so I would expect that anyway, but one needs to know in order to interpret what has been done.

I was also a little disappointed that rather than pursuing one gene/target site in some more depth to gain insight into a developmental mechanism, the authors target different tissues, different stages and different genes and each phenotype is described relatively superficially. Given that the technical advance is mainly about using a single targeting construct delivered by the RCAS vector, and that in most of the cases presented the findings are mainly confirmatory of other knockouts of these genes in other systems, there is also little new biological information.

© 2021. Published by The Company of Biologists under the terms of the Creative Commons Attribution License (https://creativecommons.org/licenses/by/4.0/).

Because there are now several other studies demonstrating real K/O or other genome engineering events using CRISPR-Cas9 technology in avian embryos, the innovation here is mainly the design of the vector and its modifications to improve efficiency. So I would strongly encourage the authors to use their new approach to follow cell lineages (ie descendants of SINGLE marked progenitor cells) for which there is great desire in the field.

More minor comments/questions:
1. I thought that a given cell can only be infected by a single RCAS particle (and this is presumably why the authors state that they use "pathogen free" embryos. Here co-injection of a high concentration of a different virus carrying a Citrine reporter is used as "efficiency control" for infection. Is this really a good control? Presumably one is seeing different populations of cells targeted by one or the other virus.

2. There is very little information about how many repeats were done of each experiments, how many were successful or the range of results seen, etc. yet some statements in the text suggesting that in some cases the infection was not as successful.

3. Some spelling etc. mistakes throughout need attention - for example "envelop" is the verb, the corresponding noun is "envelope" (eg. Fig. 1 legend, page 3, etc.).

Reviewer 3

Advance summary and potential significance to field

The manuscript by Ghandi and colleagues from the Bronner lab assesses the use of replication incompetent avian (RIA) retrovirus for the purpose of tracing infected cells during chick embryo development. The authors have engineered RIA as a vehicle to deliver gRNA together with CAS9 tethered to Citrine via a self-cleaving peptide. They first test the functionality of the construct using electroporation of a single plasmid into gastrula stage chick embryos before moving into RIA. Citrine and Cas9 were detected in tissues and the release of a functional gRNA molecule was validated indirectly, by confirming the loss or reduction of the targeted gene of interest, the transcription factors Sox10, Pax6 and Pax7, using immunohistochemistry. The ability of mutant cells to differentiate in the context of their normal environment is assessed. Furthermore they show that it is possible to observe mutant cells in real time using slice cultures derived from electroporated embryos where trunk NCCs were targeted. As an example, they confirm that loss of Sox10 leads to aberrant migration of NCCs.

Overall the approach described is novel and it will be of interest to researchers investigating cell fate specification and tissue differentiation using the chick model. The authors have validated the approach and illustrate its application in a number of different areas of developmental biology. However, at present the technique is not described in sufficient detail to be easily replicated in other laboratories and this should be addressed.

Comments for the author

The authors show that, using high titer virus, different tissues in avian embryos can be infected. This would benefit from a detailed time-course of when Cas9 and citrine proteins can first be detected. The authors comment that 24 hours was insufficient, and that proportion of cells expressing citrine increased over time.

However, this increase over time needs to be better characterized. This is particularly relevant as the authors claim that the RIA approach mirrors inducible systems in other model organisms (line 275). Furthermore, in the context of targeting Pax6 in the eye, they also mention the delay between infection and successful gene targeting and this is meant to mimic a conditional inactivation of the gene at a particular time. The delay and the temporal knockdown mediated by RIA-CRISPR needs to be demonstrated more comprehensively
They show that cells which express citrine, and thus CAS9 and a gRNA, have lost detectable expression of the targeted gene of interest. They show this with a few examples, Sox10 in migratory cranial crest and trunk crest cells, Pax6 in the retina and Pax7 in presomitic mesoderm.

© 2021. Published by The Company of Biologists under the terms of the Creative Commons Attribution License (https://creativecommons.org/licenses/by/4.0/).

Separate channels should be shown for some of the images (see below). RIA mediated infection leads to labelling of few cells, which enables them to examine genome edited cells, and their behaviour, in the context of wild-type neighbours in a mosaic tissue.

In some of the RIA infected late stage embryos Citrine positive cells were largely negative for the targeted gene (Sox10, Pax6). However, some Citrine positive cells still expressed Sox10/Pax6. The authors should comment on the possibility that the relevant cell populations (NCC for Sox10, or retina cells for Pax6) might actually be heterogeneous for expression of the transcription factor, with some cells switching off its expression at later stages in development. Therefore, a 'negative' readout may not necessarily be due to gene KO.

Additional minor improvements:
All gRNA sequences used should be given in the Materials & Methods section. The authors should indicate how relevant plasmids, including RIA, will be made available to the community.

61/62 "viability of electroporated embryos harvested at later stages is low." This would benefit from a better explanation for the non-expert reader. (line 402) For the presegmented mesoderm the authors state that electroporation can reduce viability. To better contrast this with their improved approach the number and proportion of RIA-injected embryos surviving 3-4 days post-infection should also be included. This could be added to the methods section, e.g. the authors comment on incubation for 3-5 days following virus injection (line 502).

405 It is not clear whether RIA-CFP is always included as an infection control. The methods section should include the proportions of RIA-CFP and RIA-CRISPR that are used for infection. The authors should comment on double infection of cells using these viruses, are they different sub-types?

129-137 Generation of high titer virus stock is crucial for this approach to be successful. The authors briefly describe the principle changes made to optimize this protocol, but details are missing. The protocol (478-497) should include all necessary detail to reproduce the method.

192 "in all well-transfected embryos" It is not clear how transfection was assessed in the example shown here as there is no plasmid tracer co-expressed. Can the authors comment on the variability of transfection efficiency and what efficiency is required to observe the desired knock-down? Related to this:

510 what proportion of embryos are poorly transfected, how many embryos are used for a typical experiment and 471 "embryos were screened for transfection efficiency" Authors should explain briefly how the screening is done.

261 "and did not undergo premature apoptosis". It is not clear that the authors examined whether some Sox10 KO cells undergo apoptosis. Therefore, this statement is not supported as it cannot be excluded that some cells do apoptose. Accordingly, the conclusion should be amended (line 274 "maintaining neural crest cell viability").

586 (Fig. 1 legend) how did they confirm that staining is in the nucleolus specifically, the text (174) refers to nuclear Cas9

Fig. 2 B-D, no bright field image is shown for the HH9 embryos, please comment on whether the view is similar to that shown in panel E for the HH10 embryo. For panels G-P add the times shown.

308/319/321/342/353/359 refers to Figure 3B-D, 3E and 3E', 3H etc. respectively, this should be Figure 4

Fig. 4I It is difficult to see the Citrine+/ Isl1+ double positive cells and separate channels should be shown.

Similarly in 4J, separate channels will identify double or single positive cells.

645 Fig. 5 The legend should be amended to better reflect what is shown, as the knockdown does not appear to be that efficient and Snail2 is indirectly affected.

© 2021. Published by The Company of Biologists under the terms of the Creative Commons Attribution License (https://creativecommons.org/licenses/by/4.0/).

656 It is not clear what is meant by “several Pax7 positive cells fail to specify…..” is this a typo? Furthermore the panels D-D” are very small and this data should be properly quantified to support the statement in the text (line 396) “we observed a significant reduction in the expression of Snail2”.

Fig. 5K The text states that Pax7 mutant cells expressed strong levels of MF20, however this is difficult to see and separate channels should be shown.

Fig. 5M Without further characterization the statement that cells negative for MF20 expression correspond to muscle satellite cell precursors should be removed (line 427/428).

First revision

Author response to reviewers' comments

Reviewer 1

General Comment:

To fully capitalize on the potential of the reported tools, significant quantification must be added to the figures and text to provide support for the statements made and ensure validity behind the claim of robustness in labelling and genome editing. Throughout the manuscript many of the claims made are based on a single image shown in the figure and need quantification

We thank the reviewer for their constructive feedback on our manuscript. In this revised version, we have added quantitation throughout as requested and detailed below.

1. Fig1E-F – quantify number of co-infected cells compared to total to claim significant reduction. Additionally, no mention of number of embryos compared, need n values

We thank the reviewer for this comment. While it is true that this experiment was performed by injecting two different viruses (RIA-nls-Cas9-nls and RCAS-U6-Sox10gRNAf+e) in the lumen of the neural tube, SOX10 expression was not affected in cells infected with the Cas9 virus. Moreover, we did not have a way to identify cells that were co-infected with the two viruses, as the RCAS-U6-Sox10gRNAf+e virus lacked a fluorescent reporter. We believe that this approach did not work due to poor co-infection with the two viruses. However, given the confusion this may have caused, we have removed these data from the revised manuscript.

2. - Fig 2 – quantification of *in situs* and of cell numbers and embryo numbers throughout figure

We thank the reviewer for pointing out this oversight. Given that chromogenic in situs cannot be quantified appropriately, we have used high resolution in situ hybridization chain reaction (HCR) to label Sox10 transcripts in knockout embryos. HCR allows quantification of signal intensity, which is now included in the revised. The number of embryos used for the quantification is now also mentioned.

3. Fig 3 - “As expected, Pax6 expression was missing in most of the Citrine+ cells [Figure 3H],” needs quantification.

Quantification has been added as requested.

4. Fig 4H,I,J – the text often states “mostly” in reference to either and increase or decrease of fluorescence. This should be quantified. Number of embryos examined needs to be reported.

© 2021. Published by The Company of Biologists under the terms of the Creative Commons Attribution License (https://creativecommons.org/licenses/by/4.0/).

Thank you--the quantification is now included in the revised manuscript.

5. Fig 5B-D - quantification of reduction in expression needed to claim significance

We have quantified the effect of losing Pax7 on neural crest migration area, number of neural crest cells, and PAX7 fluorescence intensity, which are now included in the revised manuscript.

Minor comments:

1. In the text the authors claim, "Together, these results confirm that the Sox10 gRNA molecules were successfully synthesized in transfected cells, and that cranial neural crest migration is inhibited in the absence of Sox10". This claim would benefit from a co-stain for the SOX10 protein and presence of Cas9 showing both lack of endpoint movement coordinated with loss of expression of SOX10. Currently, the broad in situs are not convincing to this end. Additionally, move this conclusion phrase to after the live imaging data is presented.

We thank the reviewer for this comment. Others, including us, have previously shown that Sox10 is required for proper neural crest migration. The studies that described this phenotype were cited in lines 228-231 of our original submission. The migration defect was only used as a proxy for a successful Sox10 knockdown, and we separately validated the knockdown at both the mRNA and protein levels. To address the reviewer's concern, this section of the manuscript has been rephrased.

2. In Fig 2G-P what does the dotted line refer to?

We thank the reviewer for pointing out the confusion in our figure panels. The dotted line refers to the position of the neural tube and the notochord in subsequent panels. We now clarify this in the figure legend.

3. Include the timesteps in the figure legend.

The timesteps have now been added to the figure panels.

4. Additionally, why is there no RFP displayed in panels G-K when the text reports that the control embryos had RFP-H2B expression?

This was an oversight on our part. The control embryos were not electroporated with the nuclear RFP construct, contrary to how it was described in our original submission. This has now been fixed in the revised manuscript.

5. In Fig 2G-P, because of the separate frames needed to display the time-lapse, it would be beneficial to show labelled cell tracks to better visualize and quantify the claims made about migration.

Good idea. Accordingly, we have added figure panels with cell trajectories corresponding to the cells pointed to with arrowheads.

6. what HH stage are the embryos in Fig 3?

The embryos referred to in figure 3 are embryonic day 4. We have added this information in the revised figure legend.

7. The authors reference a delay in Citrine mRNA expression which correlates with a delay in KO, a quantification of this delay would be beneficial.

We thank the reviewer for this comment. To address this delay, we performed additional experiments using the chicken DF1 fibroblast cell line. We infected DF1 cells with the RIA-CRISPR retrovirus, collected cells at 24 hours, 48 hours, and 72 hours post-infection, and

© 2021. Published by The Company of Biologists under the terms of the Creative Commons Attribution License (https://creativecommons.org/licenses/by/4.0/).

processed them for immunohistochemistry. Citrine expression was first observed in cells collected at the 48 hour timepoint, validating our original hypothesis that there is a delay between infection and expression. We also quantified this effect and report the findings as part of Figure 5 of the revised manuscript.

8. Because the guides used for SOX10 and PAX7 have already been developed, it is confusing when the details on the sequence and binding location of the PAX6 guide are highlighted in the text and figures. The authors should make it very clear in the main text that the other guides are taken from previously published studies.

We thank the reviewer for this recommendation. While this was described in line 480 of our original manuscript, we have now added this detail in the main text of the revised manuscript.

9. Line 406 of the main text switches from terming embryos in HH stages to E4.5. which is confusing for the reader. Please provide consistent or expanded explanation.

We thank the reviewer for this comment. We have now used the Hamburger-Hamilton (HH) staging method to term embryos through the revised manuscript.

Reviewer 2

General Comment:
Although the title of this manuscript and the abstract promise a method for lineage analysis based on replication-incompetent retroviral delivery of CRISPR-Cas9 constructs, the bulk of this paper is focused on generating presumed knock-outs using this technique. From this point of view, the advance is relatively minor, since several other groups around the world have already shown convincing loss of function using CRISPR- Cas9 in avian embryos (chick and quail) targeting various genes both to somatic and germ line cells, in some cases also using various viral vectors. Because there is great need in the field for this, I was hoping that the combination of RCAS as a replication incompetent vector, with the opportunity to express different combinations of labels (perhaps made more diverse using rare recombination events, or scarring, or combinatorial events) giving rise to multiple tags allowing entire lineage trees to be followed. Unfortunately despite the focus of the abstract and introduction and to some extent the title, this paper does not contain this.

We thank the reviewer for their comments and valuable feedback making us realize that the original version of our manuscript did not adequately describe the novelty of our approach, which is two-fold. The biggest and most useful advance in this study is the ability to use a single construct for concomitant delivery of Cas9, guide RNAs, and a fluorescent reporter. An issue with previously published techniques, including our own, was the need to simultaneously electroporate multiple constructs into the same cell and at the same axial level. There was no guarantee that a cell labeled with the fluorescent protein would have received both Cas9 and guide RNAs, and therefore, it was difficult to assess which cells were true "mutants". Our single-plasmid technique circumvents this issue by permitting delivery of all reagents via electroporation of a single construct. The second major advance is incorporating this single-plasmid into a retroviral construct which enables integration into the genome and the ability to lineally follow mutant cells in a normal background.
In the revised manuscript, we have extensively reorganized the manuscript and also changed the title and abstract to better explain these advantages. We have added experiments that demonstrate the proof-of- principle for using our CRISPR retroviruses for the application of clonal analysis. To do this, we combine our RIA-CRISPR viruses with retroviruses encoding nuclear RFP to label and follow clonally related cells within the same embryo. We have also included additional experiments that demonstrate the versatility of our single- plasmid approach by targeting ß-catenin in chick DF1 and human U2OS cell lines using the same construct. We believe that the revisions made to this manuscript have addressed the concerns raised by the reviewer and has therefore expanded the scope of our paper.

1. For a paper that focuses so strongly on gene knockout, in my opinion it is essential to

© 2021. Published by The Company of Biologists under the terms of the Creative Commons Attribution License (https://creativecommons.org/licenses/by/4.0/).

show that such a knockout has actually occurred. In this case, the only evidence provided that target genes like Sox10, Pax6 and Pax7 have indeed been edited out of the genome is the apparent absence of fluorescence using antibody staining. There could be several possible reasons for lack of expression including effects on transcription and/or translation or other issues, and to make strong definitive statements like "... demonstrating that the Sox10 gene and protein were deleted" (p 10, lines 257-259), or more problematically "... we observed several citrine-positive MUTANT cells distributed around the retina, suggesting that Pax6 was NO LONGER REQUIRED for the survival of these cells." (p. 12, lines 331-332) really requires direct demonstration of the editing event, at least using genomic PCR with specific primers, or better by sequencing targeted cells (although this may be difficult given the mosaic nature of the transfection and the likely multiple/diverse targeting events).

We thank the reviewer for their comment. While we agree that direct demonstration of the editing event would be the most definitive form of evidence to support claims regarding gene editing, and in an ideal world, we would love to do as the reviewer suggests and identify the editing event in each cell, neither is practical or feasible. The genome of individual cells will be uniquely edited, and we cannot think of an approach that would show this editing event in clonally related cells. As the reviewer rightly points out, it is not possible to isolate labeled cells from embryos infected with the RIA-CRISPR retroviruses because of sparse labeling observed in these embryos. The goal of this analysis is to knock-down the gene of interest. Showing absence of protein or transcript gives us the confidence that this has worked as expected. Moreover, we (including several others) have previously demonstrated a strong correlation between loss of mRNA/protein and editing events using sequencing-based approaches. Therefore, to address this concern, we have softened the language in the text (including excerpts highlighted by the reviewer in their comment above) of the revised manuscript.

2. But I think this is really important for a paper like this to reach the conclusions presented - this is even more important because there are examples in the paper where the results are not quite what is expected based on this interpretation, for example "a couple of cells" in some cases that clearly have been targeted but do have expression of the supposedly knocked-out gene. Presumably in a finite number of cases only one of the alleles will have been deleted, so I would expect that anyway, but one needs to know in order to interpret what has been done.

We thank the reviewer for this comment. We absolutely agree that editing of a single allele is a possibility, one that would just as likely yield a false negative sequencing-based result. However, given that it is a shortcoming of the method, we have added discussion addressing this possibility throughout the manuscript.

3. I was also a little disappointed that rather than pursuing one gene/target site in some more depth to gain insight into a developmental mechanism, the authors target different tissues, different stages and different genes and each phenotype is described relatively superficially. Given that the technical advance is mainly about using a single targeting construct delivered by the RCAS vector, and that in most of the cases presented the findings are mainly confirmatory of other knockouts of these genes in other systems, there is also little new biological information.

We respectfully disagree with the reviewer on this comment. Our manuscript was submitted as a “Techniques and Resource” article, where our goal was to describe: (1) modifications made to the current plasmid-based CRISPR delivery system, and (2) new set of retroviral tools for long-term labeling, editing, and clonal analysis in later-stage embryos. To ensure the efficacy of our modified reagents, it was important to show their application in a variety of different contexts. As a result, we chose to intentionally target genes about which much was known so that we could confirm the findings using our novel approach. Our choice to go “broad” rather than “deep” allowed us to illustrate how these reagents can be applied to diverse embryonic systems and later stages of development. We hope that this technique will be of great use to the chick community for further exploratory studies. However, our goal was to show that the first application works and is applicable to multiple tissues.

© 2021. Published by The Company of Biologists under the terms of the Creative Commons Attribution License (https://creativecommons.org/licenses/by/4.0/).

4. Because there are now several other studies demonstrating real K/O or other genome engineering events using CRISPR-Cas9 technology in avian embryos, the innovation here is mainly the design of the vector and its modifications to improve efficiency. So I would strongly encourage the authors to use their new approach to follow cell lineages (i.e. descendants of SINGLE marked progenitor cells) for which there is great desire in the field.

We thank the reviewer for this suggestion. To address this, we have performed clonal analysis in embryos that were injected with a combination of viruses to label mutant cells in the neural tube. The combinatorial labeling allowed us to identify of double-labeled clonally-related mutant daughter cells in the neural tube. These results of this experiment are now included in the revised manuscript.

Minor comments/questions:

1. I thought that a given cell can only be infected by a single RCAS particle (and this is presumably why the authors state that they use "pathogen free" embryos. Here co-injection of a high concentration of a different virus carrying a Citrine reporter is used as "efficiency control" for infection. Is this really a good control? Presumably one is seeing different populations of cells targeted by one or the other virus.

We thank the reviewer for pointing out this confusion. Our original approach to implement retroviral delivery of CRISPR reagents relied on using RIA and RCAS vectors. However, the results of those experiments were negative, probably due to low probability of co-infection. An efficiency control was necessary to ensure that the embryos were successfully labeled, as it was challenging to identify Citrine+ cells on embryonic day 4 or later in wholemount embryos. A single cell can be infected with multiple RIA viruses, although the probability of coinfection is low. However, the nature of these infrequent coinfections allowed us to perform lineage tracing and identify clonally-related cells, the results of which are now presented in Figure 5 of the revised manuscript.

2. There is very little information about how many repeats were done of each experiments, how many were successful or the range of results seen, etc. yet some statements in the text suggesting that in some cases the infection was not as successful.

We thank the reviewer for pointing out this oversight. Accordingly, we have now added quantification data throughout the manuscript.

3. Some spelling etc. mistakes throughout need attention - for example "envelop" is the verb, the corresponding noun is "envelope" (e.g. Fig. 1 legend, page 3, etc.).

We thank the reviewer for pointing out this mistake. It has been corrected in the revised manuscript.

Reviewer 3

General Comments:

Overall the approach described is novel and it will be of interest to researchers investigating cell fate specification and tissue differentiation using the chick model. The authors have validated the approach and illustrate its application in a number of different areas of developmental biology. However, at present the technique is not described in sufficient detail to be easily replicated in other laboratories and this should be addressed.

We thank the reviewer for their constructive feedback on our manuscript. In this revised version, we have addressed these concerns by providing more detail that will aid in replication of our results, which we agree strengthens the manuscript. We have also provided supplementary protocols for cloning the single- plasmid constructs and synthesizing viruses using chick DF1 fibroblast cell line.

© 2021. Published by The Company of Biologists under the terms of the Creative Commons Attribution License (https://creativecommons.org/licenses/by/4.0/).

Specific Concerns:

1. The authors show that, using high titer virus, different tissues in avian embryos can be infected. This would benefit from a detailed time-course of when Cas9 and citrine proteins can first be detected. The authors comment that 24 hours was insufficient, and that proportion of cells expressing citrine increased over time. However, this increase over time needs to be better characterized. This is particularly relevant as the authors claim that the RIA approach mirrors inducible systems in other model organisms (line 275). Furthermore, in the context of targeting Pax6 in the eye, they also mention the delay between infection and successful gene targeting and this is meant to mimic a conditional inactivation of the gene at a particular time. The delay and the temporal knockdown mediated by RIA- CRISPR needs to be demonstrated more comprehensively.

We thank the reviewer for this comment and agree that a detailed time course would be very useful. To address this delay, we performed additional experiments using the chicken DF1 fibroblast cell line. We infected DF1 cells with the RIA-CRISPR retrovirus, collected cells at 24 hours, 48 hours, and 72 hours post-infection, and processed them for immunohistochemistry. Citrine expression was first observed in cells collected at the 48 hour timepoint, validating our original hypothesis that there is a delay between infection and expression. We also quantified this effect and report the findings as part of Figure 5 of the revised manuscript.

2. They show that cells which express citrine, and thus CAS9 and a gRNA, have lost detectable expression of the targeted gene of interest. They show this with a few examples, Sox10 in migratory cranial crest and trunk crest cells, Pax6 in the retina and Pax7 in presomitic mesoderm. Separate channels should be shown for some of the images (see below).

We thank the reviewer for this suggestion. We have now included single channel images in figure panels as necessary.

3. RIA mediated infection leads to labelling of few cells, which enables them to examine genome edited cells, and their behavior, in the context of wild-type neighbors in a mosaic tissue. In some of the RIA infected late stage embryos Citrine positive cells were largely negative for the targeted gene (Sox10, Pax6). However, some Citrine positive cells still expressed Sox10/Pax6. The authors should comment on the possibility that the relevant cell populations (NCC for Sox10, or retina cells for Pax6) might actually be heterogeneous for expression of the transcription factor, with some cells switching off its expression at later stages in development. Therefore, a 'negative' readout may not necessarily be due to gene KO.

We thank the reviewer for this important issue. Accordingly, we now comment on the possibility that indeed some of these tissues may be heterogeneous for the expression of the targeted protein. In addition, we also discuss the possibility that in some cells, only one allele may be deleted which could also explain sustained expression of the targeted protein in infected cells.

Minor Comments

1. All gRNA sequences used should be given in the Materials & Methods section. The authors should indicate how relevant plasmids, including RIA, will be made available to the community.

We thank the reviewer for this suggestion. All plasmids are being submitted to Addgene, where they will be available for the entire community. The gRNA sequences are now mentioned in the Materials and Methods section.

2. 61/62 "viability of electroporated embryos harvested at later stages is low." This would benefit from a better explanation for the non-expert reader. (line 402) For the presegmented mesoderm the authors state that electroporation can reduce viability. To

© 2021. Published by The Company of Biologists under the terms of the Creative Commons Attribution License (https://creativecommons.org/licenses/by/4.0/).

better contrast this with their improved approach the number and proportion of RIA-injected embryos surviving 3-4 days post-infection should also be included. This could be added to the methods section, e.g. the authors comment on incubation for 3-5 days following virus injection (line 502).

We thank the reviewer for this suggestion. It has now been added to the revised manuscript text (line 590).

3. 405 It is not clear whether RIA-CFP is always included as an infection control. The methods section should include the proportions of RIA-CFP and RIA-CRISPR that are used for infection. The authors should comment on double infection of cells using these viruses, are they different sub-types?

We thank the reviewer for pointing out this confusion. RIA-CFP was not used as an infection control for all experiments. In some cases, a membrane-RFP virus was used. The proportion of cells infected with the RIA-CRISPR virus was consistently low, reflecting the differences in the virus titers. This is now better explained in the revised manuscript within the clonal analysis section.

4. 129-137 Generation of high titer virus stock is crucial for this approach to be successful. The authors briefly describe the principle changes made to optimize this protocol, but details are missing. The protocol (478-497) should include all necessary detail to reproduce the method.

We thank the reviewer for their comment. A detailed supplementary protocol is now included with the revised manuscript.

5. 192 "in all well-transfected embryos" It is not clear how transfection was assessed in the example shown here as there is no plasmid tracer co-expressed. Can the authors comment on the variability of transfection efficiency and what efficiency is required to observe the desired knock-down? Related to this:510 what proportion of embryos are poorly transfected, how many embryos are used for a typical experiment and 471 "embryos were screened for transfection efficiency" Authors should explain briefly how the screening is done.

We thank the reviewer for pointing out this confusion. Screening embryos for transfection is a common practice in the chick neural crest research community. One of the advantages of our single-plasmid approach was the presence of the Citrine fluorescent protein in all electroporated embryos. Only embryos that were morphologically normal and had high expression of Citrine within the neural tube (as witnessed in whole-mount embryos) were selected for subsequent analysis. We have now added details on how the screening was done in the Materials and Methods section. The proportion of poorly transfected embryos is highly variable and dependent of egg quality for that particular day, which is out of our control. When we make cross-sections through electroporated embryos, transfected cells are usually negative for the gene that was targeted. However, for a prominent migration defect, 75-80% of the dorsal neural tube should be transfected. The quantification for each experiment is now also included in the main text.

6. 261 "and did not undergo premature apoptosis". It is not clear that the authors examined whether some Sox10 KO cells undergo apoptosis. Therefore, this statement is not supported as it cannot be excluded that some cells do apoptose. Accordingly, the conclusion should be amended (line 274 "maintaining neural crest cell viability").

We thank the reviewer for pointing out this confusion. Previous studies have demonstrated the role of Sox10 or lack thereof in neural crest apoptosis. In our live imaging experiment, most of the transfected cells in the control embryos migrated normally, as compared to the Sox10 knockout group, where transfected cells underwent apoptosis. In the revised manuscript, we have added discussion for the possibility that some cells may undergo spontaneous apoptosis.

© 2021. Published by The Company of Biologists under the terms of the Creative Commons Attribution License (https://creativecommons.org/licenses/by/4.0/).

7. 586 (Fig. 1 legend) how did they confirm that staining is in the nucleolus specifically, the text (174) refers to nuclear Cas9

We thank the reviewer for this comment. When embryos transfected with a Cas9 expression construct without a gene-specific gRNA are stained for Cas9 protein, the signal appears as two bright dots inside the nucleus, which corresponds to the nucleolus. This has been documented by other labs before us. To address the reviewer's comment, we now include appropriate citations in the revised manuscript.

8. Fig. 2 B-D, no bright field image is shown for the HH9 embryos, please comment on whether the view is similar to that shown in panel E for the HH10 embryo.

We thank the reviewer for pointing out this confusion. We have replaced the embryo at HH10 with an earlier-staged embryo. Moreover, in several places throughout the figures, representative brightfield images are now included as inset panels.

9. For panels G-P add the times shown.

The timesteps have now been added to the figure panels.

10. 308/319/321/342/353/359 refers to Figure 3B-D, 3E and 3E', 3H etc. respectively, this should be Figure 4Fig. 4I It is difficult to see the Citrine+/ Isl1+ double positive cells and separate channels should be shown. Similarly in 4J, separate channels will identify double or single positive cells.

We thank the reviewer for this suggestion. We have now included single channel images in figure panels as necessary.

11. 645 Fig. 5 The legend should be amended to better reflect what is shown, as the knockdown does not appear to be that efficient and Snail2 is indirectly affected. 656 It is not clear what is meant by "several Pax7 positive cells fail to specify….." is this a typo? Furthermore, the panels D-D" are very small and this data should be properly quantified to support the statement in the text (line 396) "we observed a significant reduction in the expression of Snail2".

We thank the reviewer for pointing out this confusion. Given that the effect of Pax7 knockdown on Snail2 is indirect, we have removed these data from the manuscript. Figure 8 of the revised manuscript now includes in situ Hybridization Chain Reaction data against the neural crest specifier gene FoxD3, which is directly regulated by Pax7. We have also quantified the effect and presented the data in the same figure.

12. Fig. 5K The text states that Pax7 mutant cells expressed strong levels of MF20, however this is difficult to see and separate channels should be shown.

We thank the reviewer for this suggestion. We have now included single channel images in figure panels as necessary.

13. Fig. 5M Without further characterization the statement that cells negative for MF20 expression correspond to muscle satellite cell precursors should be removed (line 427/428).

We thank the reviewer for this comment. The real identification of muscle satellite cells relies on developing embryos past E10, which is not feasible in our lab. The proximity of these cells to skeletal muscles was used as a proxy for their identification as satellite cell precursors. In the revised manuscript, we have added clarification for this point.

© 2021. Published by The Company of Biologists under the terms of the Creative Commons Attribution License (https://creativecommons.org/licenses/by/4.0/).

Second decision letter

MS ID#: DEVELOP/2020/193565

MS TITLE: A single-plasmid approach for temporally-controlled mutational and lineage analysis

AUTHORS: Shashank Gandhi, Yuwei Li, Weiyi Tang, Jens Bager Christensen, Hugo A. Urrutia, Felipe M. Vieceli, Michael L Piacentino, and Marianne Bronner

I have now received all the referees' reports on the above manuscript, and have reached a decision. The referees' comments are appended below, or you can access them online: please go to BenchPress and click on the 'Manuscripts with Decisions' queue in the Author Area.

As you will see, all three referees express considerable interest in your work. While Referee 1 is satisfied with the revisions to the study, Referees 2 and 3 have some significant criticisms and recommend a substantial revision of your manuscript before we can consider publication. Referee 3 raises several points that need to be addressed or clarified, I think these are constructive suggestions and will help strengthen your study. In addition, both Referee 2 and 3 question the conclusions of the clonal analysis approach you included in the revision. It will be important to address these criticisms

For this reason, I am designating this a "major revision". I am always very reluctant to send an already revised study back for another major revision. I would like to emphasise that we are keen to publish the study, but given the concerns of both Referees 2 and 3, which I agree with, it seems the only appropriate way forward, particularly as this is for the Tools and Resources section of the journal. I would be happy to discuss the revision with you, if that would be helpful.

If you are able to revise the manuscript along the lines suggested, which may involve further experiments, I will be happy receive a revised version of the manuscript. Your revised paper will be re-reviewed by one or more of the original referees, and acceptance of your manuscript will depend on your addressing satisfactorily the reviewers' major concerns.

We are aware that you may be experiencing disruption to the normal running of your lab that make experimental revisions challenging. If it would be helpful, we encourage you to contact us to discuss your revision in greater detail. Please send us a point-by-point response indicating where you are able to address concerns raised (either experimentally or by changes to the text) and where you will not be able to do so within the normal timeframe of a revision. We will then provide further guidance. Please also note that we are happy to extend revision timeframes as necessary.

Please attend to all of the reviewers' comments and ensure that you clearly highlight all changes made in the revised manuscript. Please avoid using 'Tracked changes' in Word files as these are lost in PDF conversion. I should be grateful if you would also provide a point-by-point response detailing how you have dealt with the points raised by the reviewers in the 'Response to Reviewers' box. If you do not agree with any of their criticisms or suggestions please explain clearly why this is so.

Reviewer 1

Advance summary and potential significance to field

THe manuscript prepared by Gandhi et. al. demonstrates an important step forward in CRISPR/Cas9 tool development in chicken embryos. As hihglighte by the authors, hey have developed a system that allows for single plasmid driven expression of both the targeting gRNA, Cas9, and a fluorescent marker for lineage tracing without relying on the chicken specific U6 promotor. This is important as it facillitates a system that can be developed and used across scpecies. The authors then go on the show knockout of genes Ctnnb1, SOX10, PAX6, an PAX7 across different embryonic stages and tissues, underlining ther versitility of the technique. Finally, they adapt this one plasmid system to viral transduction and demonstrate long term lineage tracing of cells that recieved CRISPR knock out. Overall, the findings and tools developed in this manuscript with allow for many labs in the

© 2021. Published by The Company of Biologists under the terms of the Creative Commons Attribution License (https://creativecommons.org/licenses/by/4.0/).

field of developmental biology to probe questions that were previously difficult to answer, due to the difficulty of tracing genetically manipulated cells over time.

Comments for the author

I feel that the manuscript is suitable for publication with the following minor comments.
1. Often the authors use pink and red coloring in their figures which can at time be very hard to distinguish, making it difficult to judge the validity of their claims of double positive cells or lack there of.

2. Can the authors comment on the effeciency of proper cleavage of Cad9 and Citrine? Is it garenteed that when citrine expression is observed, there will also always be Cas9 present?

3.In line 345 the authors state that will loss of SOX10 expression there is also "loss of neural crest markers" but only go on to show one such marker. Please amend this statement to reflect what is shown.

<u>Reviewer 2</u>

Advance summary and potential significance to field

A method with potential as a tool to modify the genome of somatic cells in chicken embryos, and possibly to couple this with lineage tracing/clonal analysis in vivo. If the authors can prove their claims, including necessary controls and level of analysis, the tool should become very useful in the field for a number of applications.

Comments for the author

The authors have improved various aspects of this manuscript particularly by toning down their claims in several places, removal of some of the weaker results from the first version, and some improvement in the writing. I still think that there are some aspects of the work remain sloppy, but this is now more transparent to readers who read the paper carefully. The main problems I pointed out in the original review (lack of direct verification of real knockout, sometimes using phenotypic criteria in a whole cell population to determine what had been done to individual cells, etc) remain although less obvious and slightly less over-stated than before. One additional problem but important for a paper claiming to present a new method to study cell lineages.
It is critically important to demonstrate clonality, as well as to demonstrate that the targeting events are rare enough that each group of reasonably closely located cells is a clone. There is a strong statement in the Results saying that the probability of infection in one condition is 1:10000 and in another condition 1:100000, with reference to the methods for how this was estimated. In fact the methods only present a THEORETICAL calculation of probability based on textbook assumptions of viral titer (which looks impressive with all the equations) but there is no real experimental quantification or other way to demonstrate clonality. This is so central to any claim that this is a lineage tracing technique that I think the authors do need to provide some way to prove experimentally that this method really does "what it says on the tin". Although there are several possible approaches for doing this, I do understand that this is difficult experimentally - but this is not an excuse for claiming something for which the only evidence is a set of assumptions.

I really would like to see this paper published but I am concerned that these problems can set the standard for others to follow in terms of drawing firm conclusions from experimental results. Therefore I encourage the authors to attempt at least to prove the clonality claimed in the paper in some more definitive way.

<u>Reviewer 3</u>

Advance summary and potential significance to field

I saw the previous version of the manuscript and I think that there are quite significant remaining concerns with the study. The overall goal is worthwhile for researchers using the avian system.

© 2021. Published by The Company of Biologists under the terms of the Creative Commons Attribution License (https://creativecommons.org/licenses/by/4.0/).

However, the authors make quite a few strong claims that are poorly supported. The manuscript needs more attention to detail seems a little rushed.
Given that this is for the Tools and Resources section of Development, the approach has not been rigorously tested and its usefulness is not fully demonstrated. For example, the long-term clonal analysis in later stage embryos, a stated advantage of the RIA infection, is not convincing. Furthermore, citrine detection is used as a proxy for Cas9-gRNA mediated editing. The authors claim to have 'temporal' control, but they do not show whether citrine expression and gene editing events are effective at the same time, or earlier, or later. Finally too often the loss of the marker gene that was targeted is assumed and not directly shown.

Comments for the author

I suggest to amend the title. Specifically the term “temporally-controlled” implies that the manipulation is inducible, which is not the case.

In the abstract, please remove “and across multiple species” (line 33) as in the context it sounds as if you are talking about embryos from multiple species. Also change the last sentence accordingly (line 41).

Fig. 1D is a merged image. Please clarify why there are many cells that are Cas9 positive but do not have any cytoplasmic citrine fluorescence, only cells in the dorsal neural tube in the hatched area seem to be citrine positive.

Fig. 2F-I these images need to improve. Transfection efficiency might have been too low, as there are no two transfected cells abutting. It would be clearer to measure b-cat at cell junctions as in the U2OS panels above. The authors suggest the same construct could be used in human and chick. However even though there might be some effect of the Hs-gRNA in DF1 cells, I think it is prudent to use a gRNA without any mismatches with the endogenous site to be targeted. Therefore, I am not convinced that this part should be a major aspect of the study, as claimed (lines 160-165).

Fig. 3 uses a previously established Sox10gRNA. Please summarise briefly where in the gene this targets, as this will allow the reader to better understand the findings made. For example, it is surprising that there is such a strong effect on protein (3D) when there is still quite a bit of transcript detected (3E, I). For the HCR quantification, it appears that total fluorescence was measured. It is not clear whether fluorescence signal is reduced in some of the ‘edited’ cells that do give an HCR signal. Are these the cells that were not targeted (citrine negative), or was only one allele affected? In order to demonstrate the efficiency of this single-plasmid they could do some FACS analysis to answer these questions.

At present the clonal analysis is not well documented.
Fig. 5 Results shown for RIA mediated Pax7 knockout/knockdown are not convincing. In particular the authors state (line 318)”One clone of neural tube cells with high Citrine and low RFP (outlined in white) had low PAX7 expression [Fig.5K-K’’], whereas the other adjacent clone with high RFP but low Citrine (outlined in blue) appeared to lack PAX7 expression.” If I understand correctly the gRNA (targeting Pax7) is delivered together with Citrine, RIA RFP does not contain any Cas9 or gRNA. This result needs some explanation. In addition, the authors talk about clones and clonally related cells, but the outline identifies individual cells rather than clones of cells. Also in this section (lines 312-315) the authors refer to KO of Pax7 for 5F-G, but there is no Pax7 staining shown, so loss or reduced Pax7 is only assumed. Similarly 5H-I there is no staining for Pax7 shown.

Fig. 6 Additional quantification should be added to support the versatility and efficiency of this approach. For example, in migratory NCC labelled with citrine Sox10 was diminished (6F), or a low level of SOX10 protein was detectable in a few Citrine-labeled cells [Fig.6I’; white solid arrowheads]. How many cells were counted and how many cells are in each category? Numbers of embryos have not been stated.

Fig 7 looks at the developing eye and RIA is used to KD Pax6. In 6I the authors show the efficiency of Pax6 knockdown is variable, but in subsequent panels they examine later phenotypes resulting from Pax6 “loss”. The authors assume “absence of Pax6”, which is not shown. This is not acceptable.

© 2021. Published by The Company of Biologists under the terms of the Creative Commons Attribution License (https://creativecommons.org/licenses/by/4.0/).

Fig. 8 Should explain the action of the validated Pax7 protospacer. The term “knockout” (line 484) should be used with caution as it’s not directly shown here, but assumed to occur. It is surprising that 8H, J are significant, please clarify is this was n=9 embryos for all of these. They observe a reduction of FoxD3 expression, a Pax7 target, particularly in the hindbrain (7I), was this the case in all embryos, n = ? The electroporation does not target the hindbrain, citrine is more widely distributed. Can this be clarified.

Fig. 8 limb myogenesis contains strong claims that need to be rephrased. Line 525: “Surprisingly, these mutant cells expressed strong levels of MF20 [Fig.8Q’,T,T’,U,U’], suggesting that Pax7 was dispensable for proper myogenesis when lost at the post- segmental plate stages.” Because Pax3 remains expressed it is not surprising that limb myogenesis occurs. “these mutant cells” makes it sound as if they are the very same cells shown in Q, which seem to be Citrine positive and Pax7 negative, however Q’ is an adjacent section showing different cells. line 528: “negative for MF20 expression” [Fig.8R]. This panel shows Pax7 staining not MF20 – please clarify.

Minor:
The statement (Line 358) “As the retroviral approach only labels a neural crest subpopulation,” is confusing.
There are many cells labelled with the ‘transfection control’ retrovirus-mem-RFP. This should be clarified The sentence line 57/58 makes no sense, “and therefore” do the authors mean to say: Thus, only those cells co-transfected with both plasmids were mutants, “but they are” indistinguishable from those transfected with only the fluorescent marker.
Same in line 96 “and therefore” this logic makes no sense to this reviewer Line 106: “from Cas9 by the self-cleaving 2A peptide sequence”. Make clear explicitly that a similar approach with regard to the 2A peptide, was previously developed and reported by Williams 2018 Line 146: replace “robust -catenin knockout” with knockdown, as KO was not shown conclusively, indeed b-
catenin protein was still present at cell membrane Lines 160-165: not sure about the following statement, it seems to distract from the main focus of the study.
“To our knowledge, this is the first demonstration of CRISPR-Cas9-mediated knockouts in two different species using the exact same construct. Together, these experiments illustrate the versatility of our single-
plasmid approach in performing perturbation experiments across multiple species, and demonstrate an important application for efficient screening of gRNA constructs across species, especially when regions with high homology near the PAM site are targeted.”
Line 184: It cannot be directly shown which and how many cells do indeed have a true knockout, therefore replace “knockout embryos” with electroporated or targeted embryos.
Line 188: similarly, replace “Sox10 loss” with reduced Sox10 Line 224: remove “normal” before “mitosis”, as without detailed quantification this has not been shown here.
Similarly, rephrase what is being stated regarding apoptosis, describe what you actually see and what leads you to suggest there is apoptosis, then say that this is consistent with previous work. Please comment on why the cells with fragmented nuclei are citrine negative.
Fig. 6 add arrows to subpanels to indicate the same cells in (E’, E”, G’, G”, I’, I”)
Fig. 7E Please clarify how the quantification was done. It seems this was done on only 4 sections, was this reproduced in multiple embryos? The control in 7F is meant to show normal development despite large insert size, however these control constructs have smaller inserts, so it is not clear how this confirms lack of interference.

Second revision

Author response to reviewers' comments

Reviewer 1

Minor Comments

© 2021. Published by The Company of Biologists under the terms of the Creative Commons Attribution License (https://creativecommons.org/licenses/by/4.0/).

1. Often the authors use pink and red coloring in their figures which can at time be very hard to distinguish, making it difficult to judge the validity of their claims of double positive cells or lack thereof.

Thank you for bringing this to our attention. To address this, we provide single-channel images to guide the reader through any data that calls attention towards double-positive cells.

2. Can the authors comment on the efficiency of proper cleavage of Cas9 and Citrine? Is it guaranteed that when citrine expression is observed, there will also always be Cas9present?

To address this point, we show that Cas9 and Citrine proteins are present and overlap in transfected cells. Indeed, Citrine folding would not be feasible unless the Cas9 and Citrine peptides are cleaved properly. In our paper, Citrine+ cells co-express Cas9, so we believe that the 2a peptide-mediated cleavage is efficient. Importantly, Williams et al 2018 previously used the Cas9-2a-Citrine system in their paper published in Development, and they have since used it in multiple publications from their group.

3. In line 345 the authors state that will loss of SOX10 expression there is also "loss of neural crest markers" but only go on to show one such marker. Please amend this statement to reflect what is shown.

We agree with the reviewer's concern and have amended this statement.

Reviewer 2

5. The authors have improved various aspects of this manuscript particularly by toning down their claims in several places, removal of some of the weaker results from the first version, and some improvement in the writing. I still think that there are some aspects of the work remain sloppy, but this is now more transparent to readers who read the paper carefully.

Despite the challenges of working during a pandemic, we added a great deal of additional data in the revised manuscript, including experiments directly addressing this reviewer's previous concerns. We are not sure what the reviewer is referring to when he/she mentions that aspects are "sloppy" and don't find this comment to be constructive.

6. The main problems I pointed out in the original review (lack of direct verification of real knockout, sometimes using phenotypic criteria in a whole cell population to determine what had been done to individual cells, etc) remain although less obvious and slightly less over-stated thanbefore.

As mentioned in our previous response, it is not feasible to sequence the mutant cells in vivo. *Thus, assessing loss of the target protein by antibody staining is the best available proxy for knockout. Moreover, given that RIA viruses sometimes edit only one allele, staining for protein is a better way to look for true null cells. We have altered the text in the revised manuscript to make this more clear.*

7. One additional problem but important for a paper claiming to present a new method to study cell lineages. It is critically important to demonstrate clonality, as well as to demonstrate that the targeting events are rare enough that each group of reasonably closely located cells is a clone. There is a strong statement in the Results saying that the probability of infection in one condition is 1:10000 and in another condition 1:100000, with reference to the methods for how this was estimated. In fact the methods only present a THEORETICAL calculation of probability based on textbook assumptions of viral titer (which looks impressive with all the equations) but there is no real experimental quantification or other way to demonstrate clonality. This is so central to any claim that this is a lineage tracing technique that I think the authors do need to provide some way to prove experimentally that this method really does "what it says on the tin". Although there are several possible approaches for doing this, I do understand that this is difficult experimentally - but this is not an excuse for claiming something for which the only evidence is a set of assumptions.

We appreciate the reviewer's concern but respectfully disagree that calculating the theoretical

© 2021. Published by The Company of Biologists under the terms of the Creative Commons Attribution License (https://creativecommons.org/licenses/by/4.0/).

probability of double and triple infection is not a reasonable way to predict clonality. In addition, because the integration site is unique for each clone, similar levels of fluorescent protein expression also helps confirm clonality. We have previously published the methodology regarding the use of avian retroviruses for clonal analysis (see Tang et al., 2019, *Dev Bio*). We have also used this method on vagal neural crest cells in our upcoming paper that is in press in *Nature Communication*. The low probability of co-infection with two viruses can be modeled using a Poisson distribution, as we have detailed in our materials and methods. While sequencing-based approaches work *in vitro* for clonally related cells, sequencing cannot be applied to our *in vivo* system due to the small number of progeny that can only be detected in sections. So we are unsure of what other piece of experimental data the reviewer would think are appropriate. As the reviewer rightly points out, such experiments are extremely difficult and not yet feasible in this *in vivo* context.
We have now modified the text to call the clones we identify "putative" clones. However, one must keep in mind that lineage analysis and clonal analysis are not necessarily synonymous. This approach works well to follow lineage at the population level and can also be applied to the single cell level by using limiting dilutions of virus or multiple infections.

8. I really would like to see this paper published but I am concerned that these problems can set the standard for others to follow in terms of drawing firm conclusions from experimental results. Therefore I encourage the authors to attempt at least to prove the clonality claimed in the paper in some more definitive way.

Performing clonal analysis with the RIA-CRISPR retrovirus results in very few clones per embryo because of the low viral titers. Thus, one can also use "sparse labeling" using limiting dilutions of virus to establish clonal identity in addition to calculating theoretical probabilities. Moreover, as stated above, lineage analysis does not only refer to clonal analysis, but one can also use this approach to follow populations of cells arising from a particular location. This is particularly well-suited for neural crest cells that start within the neural tube but then migrate extensively in the periphery. So this advantage goes far beyond clonal analysis as we can follow the lineage of labeled mutant neural crest cells regardless of whether or not they are clonally related. To address the reviewer's point, we have changed the emphasis in the text to focus on lineage analysis rather than clonal analysis.

Reviewer 3

1. *I saw the previous version of the manuscript and I think that there are quite significant remaining concerns with the study. The overall goal is worthwhile for researchers using the avian system. However, the authors make quite a few strong claims that are poorly supported. The manuscript needs more attention to detail, seems a little rushed.*

We thank the reviewer for their comments. As is pointed out below, many of the concerns raised by this reviewer stem from a misunderstanding of our figures/data which we now clarify. We have added a great deal of additional data to the revised manuscript including experiments directly addressing this reviewer's previous concerns, despite the challenges of working during a pandemic, and hope that the corrections here satisfy this reviewer.

2. Given that this is for the Tools and Resources section of Development, the approach has not been rigorously tested and its usefulness is not fully demonstrated. For example, the long-term clonal analysis in later stage embryos, a stated advantage of the RIA infection, is not convincing. Furthermore, citrine detection is used as a proxy for Cas9-gRNA mediated editing. The authors claim to have 'temporal' control, but they do not show whether citrine expression and gene editing events are effective at the same time, or earlier, or later. Finally, too often the loss of the marker gene that was targeted is assumed and not directly shown.

Our overall goal in this paper was to: (1) describe modifications made to the current plasmid-based CRISPR delivery system, which we demonstrated by:
1. *in vitro* knockdown of ß-catenin in cell lines obtained from two different species
2. *in vivo* knockdown of Pax7, Sox10, and Pax6 in early stage embryos.
3. application of our reagents for live imaging in chick embryos, a vital tool that has been missing from the toolkit until now. ; and (2) present a new set of retroviral tools for

© 2021. Published by The Company of Biologists under the terms of the Creative Commons Attribution License (https://creativecommons.org/licenses/by/4.0/).

long-term labeling, editing, and lineage analysis in later- stage embryos, which we achieved by:

4. optimizing the protocol for synthesizing viral particles containing extra-long inserts
5. tracking cells within the developing neural tube, peripheral nervous system, and the chick retina.
6. demonstrating proof-of-principle application of our viruses for clonal analysis in the chick neural tube. We believe that all these data conclusively demonstrate the usefulness of our approach, as noted by this reviewer during the first round of reviews ("…the authors have validated the approach and illustrate its application in a number of different areas of developmental biology…").

Regarding long-term clonal analysis, the technique of using avian retroviruses for clonal analysis has already published from our lab (see Tang et al., 2019, Dev Bio*), as well as in a second paper that is in press in* Nature Communications *on vagal neural crest cells. Our goal here was to establish proof-of-principle of coupling retroviral infection with gene editing, which we believe was successfully demonstrated by the experiments added in the revision process.*

We are unsure what the reviewer means when they say, "do not show whether citrine expression and gene editing events are effective at the same time, or earlier, or later." *In our revised manuscript, we showed that following infection, Citrine expression took at least 24 hours to be first observed, a trend that was followed by Cas9 as well. Given that Cas9, with its large size takes longer to be transported into the nucleus and cause double-stranded breaks, our data suggest that the editing event happens sometime after Citrine is visible. However, we wait several days after infection to collect embryos for sectioning and immunostaining, guaranteeing that Cas9 has been active for some time before analysis. Our reagents are particularly advantageous when targeting a gene that is expressed later in development or when avoiding early detrimental effects of a gene to focus on its later function.*

Regarding Citrine as a proxy for Cas9 expression, we stained for both Cas9 and Citrine and found a similar trend, showing that they are co-expressed. This suggests Cas9 activity closely correlates with Citrine expression. The 2a peptide that we use as a linker has been tried and tested in many scenarios and works very well.

Please see below for our response to the reviewer's individual comments.

3. I suggest to amend the title. Specifically the term "temporally-controlled" implies that the manipulation is inducible, which is not the case.

What we meant to imply is that our reagents offer temporal control through injection at specific developmental timepoints rather than by drug-mediated induction. This is something that cannot be achieved easily with electroporation. However, injection of the retrovirus is fairly straightforward and bypasses early effects since the Cas9 takes time to fold and become active. Inducible systems also suffer from lack of "precise" temporal control but give a good approximation of when editing events occur. That said, we understand the reviewer's concern and have changed the title accordingly.

4. In the abstract, please remove "and across multiple species" (line 33) as in the context it sounds as if you are talking about embryos from multiple species. Also change the last sentence accordingly (line 41).

We are happy to make this small edit since the focus of our paper is on the chick system. That said, by multiple species, we are referring to the data where these constructs were used to target genes in both human and chicken cell lines. We think that this strategy will be useful outside the avian developmental biology field, especially because a species-specific U6 promoter is no longer necessary to drive guide RNA expression.

5. Fig. 1D is a merged image. Please clarify why there are many cells that are Cas9 positive but do not have any cytoplasmic citrine fluorescence, only cells in the dorsal neural tube in the hatched area seem to be citrine positive.

Apologies for the misunderstanding. The red cells visible in the merged image refer to H2B-RFP-positive cells, not Cas9. Only Citrine-positive cells in the dorsal neural tube were Cas9-positive. Given the confusion this figure caused, we have removed the red (H2B-RFP) channel from the figure panel.

© 2021. Published by The Company of Biologists under the terms of the Creative Commons Attribution License (https://creativecommons.org/licenses/by/4.0/).

6. Fig. 2F-I these images need to improve. Transfection efficiency might have been too low, as there are no two transfected cells abutting. It would be clearer to measure b-cat at cell junctions as in the U2OS panels above.

Apologies for the confusion that led to the reviewer misinterpreting this figure. We now better explain the quantitation and what was observed. The human osteosarcoma cell line is epithelial, whereas the chicken DF1 cell line is mesenchymal in nature. Therefore, while the U2OS cells are often abutting, that is not the case with DF1 cells. Moreover, beta-catenin is highly expressed at cellular junctions in the U2OS cell line, whereas its expression is cytoplasmic in the DF1 fibroblast cell line. Since the U2OS cells cluster together because of their epithelial nature, it warranted a different approach for quantification, as shown in figure 2D. Transfection in DF1 cells is sparse, and since our analysis focused on single-cell measurements for beta-catenin fluorescence intensity across 108 different transfected cells, the transfection efficiency was not a confounding factor. Finally, since we are comparing the effect of using two different constructs in the DF1 cell line, we decided to use cell fluorescence intensity as a readout for efficacy, which as shown in 2I, is significantly different between the control and treatment groups.

7. The authors suggest the same construct could be used in human and chick. However even though there might be some effect of the Hs-gRNA in DF1 cells, I think it is prudent to use a gRNA without any mismatches with the endogenous site to be targeted. Therefore, I am not convinced that this part should be a major aspect of the study, as claimed (lines 160-165).

We apologize if this was not clearly stated and have revised the text and figure accordingly. Again this is a misinterpretation suggesting that we were not sufficiently clear in our explanation. In panel 2G, the gRNA with zero mismatches in the chick genome was used to target DF1 cells. In 2H, the human gRNA, which had 3 mismatches in the PAM-distal nucleotides was used to target DF1 cells. So overall, in this figure, we have demonstrated the application of the human gRNA in human cell line [Figure 2C], the chick gRNA in the chick cell line [Figure 2G], and the human gRNA in the chick cell line [Figure 2H]. All these data have been quantified in panels D and I. Figure 2 (panel E) has also been modified in response to this comment.

8. Fig. 3 uses a previously established Sox10gRNA. Please summarise briefly where in the gene this targets, as this will allow the reader to better understand the findings made. For example, it is surprising that there is such a strong effect on protein (3D) when there is still quite a bit of transcript detected (3E, I).

Again apologies for not being more clear. As noted in previous publications, the guide targets the first exon, with the PAM overlapping with the first codon of the Sox10 *coding sequence. The difference in panels 3D and 3E or 3I comes from a different stage of development. The embryo shown in 3D is premigratory (HH9), whereas the embryos in 3E and 3I are migratory stages (HH9+). There is a 1.5h difference between HH9 and HH9+. Also, it is not surprising that a small amount of transcript may be detectable, given that a neotranscript would not form a functional protein and would therefore escape immunolabeling.*

9. For the HCR quantification, it appears that total fluorescence was measured. It is not clear whether fluorescence signal is reduced in some of the 'edited' cells that do give an HCR signal. Are these the cells that were not targeted (citrine negative), or was only one allele affected? In order to demonstrate the efficiency of this single-plasmid they could do some FACS analysis to answer these questions.

Apologies for not explaining this better. Electroporation never transfects all neural crest precursors. Thus, measuring corrected total cell fluorescence (CTCF) intensity, which is a widely-accepted standard practice in the field, will measure signal in both transfected and non-transfected cells, thereby contributing to residual signal on the treated side. It is difficult to assess which cells have editing events on one or both alleles. The main message is that the single-plasmid approach works quite well for knocking down gene expression in ovo. *If anything, our quantification is an underrepresentation of the actual efficiency of the single-plasmid, but it doesn't affect the overall goal of this paper.*

© 2021. Published by The Company of Biologists under the terms of the Creative Commons Attribution License (https://creativecommons.org/licenses/by/4.0/).

10. At present the clonal analysis is not well documented. Fig. 5 Results shown for RIA mediated Pax7 knockout/knockdown are not convincing. In particular the authors state (line 318) "One clone of neural tube cells with high Citrine and low RFP (outlined in white) had low PAX7 expression [Fig.5K-K''], whereas the other adjacent clone with high RFP but low Citrine (outlined in blue) appeared to lack PAX7 expression." If I understand correctly the gRNA (targeting Pax7) is delivered together with Citrine, RIA RFP does not contain any Cas9 or gRNA. This result needs some explanation.

Apologies for this misunderstanding. The reviewer is correct that the RIA-H2B-RFP retrovirus does not contain any Cas9 or gRNA. The loss of Pax7 expression resulted from Cas9-mediated editing that was concomitant with Citrine expression in those cells, which while low in intensity, was sufficient to knockdown levels of Pax7 to a greater extent than in the neighboring clones. However, their clonal relationships are derived from double-labeling with the RIA-CRISPR and RIA-H2B-RFP retroviruses. We now explain this better in the text. We have moved the clonal analysis data to the end of the manuscript to minimize any distraction in the mind of the reader. These data are now part of Figure 9 of the revised manuscript.

11. In addition, the authors talk about clones and clonally related cells, but the outline identifies individual cells rather than clones of cells. Also in this section (lines 312-315) the authors refer to KO of Pax7 for 5F-G, but there is no Pax7 staining shown, so loss or reduced Pax7 is only assumed. Similarly 5H-I there is no staining for Pax7 shown.

Good point. We have redrawn the borders now to show clones of cells with different colors representing individual clones (white, yellow, and cyan). The goal of revised figure 5 was to show that rare clones double- labeled with the RIA-CRISPR and RIA-RFP viruses can be identified throughout the embryo, and that our reagents make it possible for developmental biology labs to target a gene necessary for the proper differentiation of neural crest cells at their terminal location and track individual clones over a long period of time. While Pax7 was a good candidate gene for its broad expression in the developing neural tube, its knockdown is inconsequential for neural crest cells that migrate away from the dorsal neural tube. This is why we believe that it was not necessary to show loss of Pax7 in the outflow tract. We have now modified the text accordingly.

12. Fig. 6 Additional quantification should be added to support the versatility and efficiency of this approach. For example, in migratory NCC labelled with citrine Sox10 was diminished (6F), or a low level of SOX10 protein was detectable in a few Citrine-labeled cells [Fig.6I'; white solid arrowheads]. How many cells were counted and how many cells are in each category? Numbers of embryos have not been stated.

Thank you for pointing out this issue. The number of embryos that were injected with the Sox10-RIA- CRISPR virus is mentioned in line 288 of the revised manuscript. Quantification is now added in the text.

13. Fig 7 looks at the developing eye and RIA is used to KD Pax6. In 6I the authors show the efficiency of Pax6 knockdown is variable, but in subsequent panels they examine later phenotypes resulting from Pax6 "loss". The authors assume "absence of Pax6", which is not shown. This is not acceptable.

Apologies for this misunderstanding. We now explain that we only had one channel for downstream immunostaining analysis, given that the embryos were injected with the RIA-CRISPR and RIA-mem-RFP retroviruses. We have modified the text to clarify accordingly.

14. Fig. 8 Should explain the action of the validated Pax7 protospacer. The term "knockout" (line 484) should be used with caution as it's not directly shown here, but assumed to occur.

Thank you for this comment. We now explain that we observed a reduction in both Pax7 fluorescence intensity and the number of Pax7-positive cells in wholemount embryos following transfection with the single plasmid. The embryos that were electroporated with this plasmid were referred to as the "knockout" group, compared to the "control" group that received the

© 2021. Published by The Company of Biologists under the terms of the Creative Commons Attribution License (https://creativecommons.org/licenses/by/4.0/).

control gRNA.

15. It is surprising that 8H, J are significant, please clarify is this was n=9 embryos for all of these. They observe a reduction of FoxD3 expression, a Pax7 target, particularly in the hindbrain (7I), was this the case in all embryos, n = ? The electroporation does not target the hindbrain, citrine is more widely distributed. Can this be clarified.

We now clarify that Figure 8H used n=9 embryos, whereas 8J used n=4 embryos. The paired student's t-test used to determine significance yielded p-values less than 0.05 for 8J, and less than 0.01 for 8H.

The electroporation covers the entire neural plate border region, which transfects cells in the dorsal neural tube along the anterior-posterior axis of the developing embryo (which includes the hindbrain). Again, this is very common in the neural crest field, and we invite the reviewer to check out our recent publication (Gandhi et al, 2020, eLife), where we carefully documented that CRISPR plasmids result in increased penetrance in the dorsal hindbrain compared to the dorsal forebrain/midbrain. We see the same effect in figure 7I. Due to limited space in the current manuscript, we are unable to expand on this phenomenon but instead cite our previous work.

16. Fig. 8 limb myogenesis contains strong claims that need to be rephrased. Line 525: "Surprisingly, these mutant cells expressed strong levels of MF20 [Fig.8Q',T,T',U,U'], suggesting that Pax7 was dispensable for proper myogenesis when lost at the post- segmental plate stages." Because Pax3 remains expressed it is not surprising that limb myogenesis occurs.

The reviewer is correct, the presence of Pax3 would result in proper myogenesis. However, that still implies that Pax7 was dispensable for myogenesis. This is well known in the literature, and we merely confirm it in our experiments. However, we take the reviewer's meaning and have toned down the statement.

17. "these mutant cells" makes it sound as if they are the very same cells shown in Q, which seem to be Citrine positive and Pax7 negative, however Q' is an adjacent section, showing different cells.

We clarify that panels 8Q and 8Q' are adjacent sections, stained for Pax7 and MF20 (we only had one channel available for immunolabeling). We believe that cells marked in 8S and 8T are the same cells, while cell marked in 8R can also be seen in 8Q'.

18. line 528: "negative for MF20 expression" [Fig.8R]. This panel shows Pax7 staining not MF20 - please clarify.

The cell shown in 8R is also visible in 8Q' and is negative for MF20 expression. We have clarified this in the text.

Minor:

19. The statement (Line 358) "As the retroviral approach only labels a neural crest subpopulation," is confusing. There are many cells labelled with the 'transfection control' retrovirus-mem-RFP. This should be clarified

Thank you for pointing out this issue. We clarify in the text that we meant few cells are labeled with the RIA-CRISPR retrovirus given its low titer.

20. The sentence line 57/58 makes no sense, "and therefore" do the authors mean to say: Thus, only those cells co-transfected with both plasmids were mutants, "but they are" indistinguishable from those transfected with only the fluorescent marker.

Thank you for pointing out that this sentence is confusing. A mutant cell may be labeled with the fluorescent marker, but it would be indistinguishable from a cell that received the fluorescent marker but not Cas9. We have fixed this in the text.

© 2021. Published by The Company of Biologists under the terms of the Creative Commons Attribution License (https://creativecommons.org/licenses/by/4.0/).

21. Same in line 96 "and therefore" this logic makes no sense to this reviewer

We have fixed this in the text.

22. Line 106: "from Cas9 by the self-cleaving 2A peptide sequence". Make clear explicitly that a similar approach,
with regard to the 2A peptide, was previously developed and reported by Williams 2018

We apologize for not making this more clear as we had to cut 2000 words from our submission to meet the word limit set by the journal. We cite their paper in lines 49, 55, 73, and 106. We do not claim that our study is the first to use a 2A peptide for expression of Cas9 and fluorescent marker.

23. Line 146: replace "robust -catenin knockout" with knockdown, as KO was not shown conclusively, indeed b- catenin protein was still present at cell membrane

This has been changed accordingly.

24. Lines 160-165: not sure about the following statement, it seems to distract from the main focus of the study. "To our knowledge, this is the first demonstration of CRISPR-Cas9-mediated knockouts in two different species using the exact same construct. Together, these experiments illustrate the versatility of our single- plasmid approach in performing perturbation experiments across multiple species, and demonstrate an important application for efficient screening of gRNA constructs across species, especially when regions with high homology near the PAM site are targeted."

We have toned down this statement.

25. Line 184: It cannot be directly shown which and how many cells do indeed have a true knockout, therefore replace "knockout embryos" with electroporated or targeted embryos.

This has been changed as requested.

14. Line 188: similarly, replace "Sox10 loss" with reduced Sox10

This has been changed.

15. Line 224: remove "normal" before "mitosis", as without detailed quantification this has not been shown here. Similarly, rephrase what is being stated regarding apoptosis, describe what you actually see and what leads you to suggest there is apoptosis, then say that this is consistent with previous work.

This has been changed.

16. Please comment on why the cells with fragmented nuclei are citrine negative.

Apologies for the misinterpretation. The cells with fragmented nuclei do in fact have Citrine expression, as seen in panel K. Eventually, a dead cell will lose its fluorescence.

17. Fig. 6 add arrows to subpanels to indicate the same cells in (E', E", G', G", I', I")

Arrows have been added to all related panels.

18. Fig. 7E Please clarify how the quantification was done. It seems this was done on only 4 sections, was this reproduced in multiple embryos?

We now clarify how this was quantitated. The four sections were taken across two different representative embryos, to validate the results in Figures 7B-D.

© 2021. Published by The Company of Biologists under the terms of the Creative Commons Attribution License (https://creativecommons.org/licenses/by/4.0/).

19. The control in 7F is meant to show normal development despite large insert size, however these control constructs have smaller inserts, so it is not clear how this confirms lack of interference.

Apologies for the misinterpretation. The size difference between the inserts contained in the virus used in 7F (RIA-nls-Cas9-nls-eGFP) and the RIA-CRISPR retrovirus is only ~200bp (4.9kb for virus in 7F versus 5.1kb for RIA-CRISPR), as has been explained in lines 266-268 of the revised manuscript.

Third decision letter

MS ID#: DEVELOP/2020/193565

MS TITLE: A single-plasmid approach for genome editing coupled with long-term lineage analysis in chick embryos

AUTHORS: Shashank Gandhi, Yuwei Li, Weiyi Tang, Jens Bager Christensen, Hugo A. Urrutia, Felipe M. Vieceli, Michael L Piacentino, and Marianne Bronner
ARTICLE TYPE: Techniques and Resources Article

I am happy to tell you that your manuscript has been accepted for publication in Development, pending our standard ethics checks.

Reviewer 3

Advance summary and potential significance to field

In this paper the authors use the chick embryo model to describe improvements to the current plasmid-based CRISPR delivery system, which they apply both in vitro using human and chick cell lines and in vivo in early stage embryos. They transfer the single vector delivery of Cas9, gRNA and citrine fluorophore to replication incompetent retrovirus (RIA). They show that viral infection can be used in later stage embryos to trace edited cells. Cells are tracked in a number of different tissues. The improved plasmid and viral vectors will be a useful addition to the tool kit available to researchers using the avian system.

Comments for the author

The authors have responded to my comments and have clarified all queries. I have no further concerns.

© 2021. Published by The Company of Biologists under the terms of the Creative Commons Attribution License (https://creativecommons.org/licenses/by/4.0/).
